# A natural gene drive system influences bovine tuberculosis susceptibility in African buffalo: Possible implications for disease management

**DOI:** 10.1371/journal.pone.0221168

**Published:** 2019-09-04

**Authors:** Pim van Hooft, Wayne M. Getz, Barend J. Greyling, Armanda D. S. Bastos

**Affiliations:** 1 Resource Ecology Group, Wageningen University, Wageningen, The Netherlands; 2 Department of Zoology & Entomology, Mammal Research Institute, University of Pretoria, Hatfield, South Africa; 3 Department of Environmental Science Policy & Management, University of California, Berkeley, California, United States of America; 4 School of Mathematical Sciences, University of KwaZulu-Natal, Durban, South Africa; 5 Agricultural Research Council, Irene, South Africa; Tokat Gaziosmanpasa University, TURKEY

## Abstract

Bovine tuberculosis (BTB) is endemic to the African buffalo (*Syncerus caffer*) of Hluhluwe-iMfolozi Park (HiP) and Kruger National Park, South Africa. In HiP, the disease has been actively managed since 1999 through a test-and-cull procedure targeting BTB-positive buffalo. Prior studies in Kruger showed associations between microsatellite alleles, BTB and body condition. A sex chromosomal meiotic drive, a form of natural gene drive, was hypothesized to be ultimately responsible. These associations indicate high-frequency occurrence of two types of male-deleterious alleles (or multiple-allele haplotypes). One type negatively affects body condition and BTB resistance in both sexes. The other type has sexually antagonistic effects: negative in males but positive in females. Here, we investigate whether a similar gene drive system is present in HiP buffalo, using 17 autosomal microsatellites and microsatellite-derived Y-chromosomal haplotypes from 401 individuals, culled in 2002–2004. We show that the association between autosomal microsatellite alleles and BTB susceptibility detected in Kruger, is also present in HiP. Further, Y-haplotype frequency dynamics indicated that a sex chromosomal meiotic drive also occurred in HiP. BTB was associated with negative selection of male-deleterious alleles in HiP, unlike positive selection in Kruger. Birth sex ratios were female-biased. We attribute negative selection and female-biased sex ratios in HiP to the absence of a Y-chromosomal sex-ratio distorter. This distorter has been hypothesized to contribute to positive selection of male-deleterious alleles and male-biased birth sex ratios in Kruger. As previously shown in Kruger, microsatellite alleles were only associated with male-deleterious effects in individuals born after wet pre-birth years; a phenomenon attributed to epigenetic modification. We identified two additional allele types: male-specific deleterious and beneficial alleles, with no discernible effect on females. Finally, we discuss how our findings may be used for breeding disease-free buffalo and implementing BTB test-and-cull programs.

## Introduction

Sexual diploid organisms are vulnerable to meiotic drivers. Meiotic drivers are selfish genetic elements that violate Mendel’s law of equal segregation by favouring their own transmission, or that of the chromosome on which they reside. They do this by distorting meiosis, or by impairing the function or increasing the mortality of the opposite-sex gametes [1]. Molecularly characterized meiotic drivers are composed of a drive locus and a closely linked sensitive responder locus, which are not always protein-coding and which are usually embedded in large recombination-free genomic regions with numerous additional loci affecting meiotic distortion [2–4]. Meiotic drivers have been reported, amongst others, in plants (*Zea mays*), insects (*Drosophila melanogaster*), fish (Actinopterygii), mammals (*Mus musculus*) and fungi (*Neurospora*) [1,5].

Sex-ratio distorters constitute a specific class of meiotic drivers that distort the primary sex ratio because they are located on one of the sex chromosomes. They have been observed in insects (Drosophilidae, Diopsidae, Tephritida, Muscidae and Culicidae), and also in mammals (*Dicrostonyx torquatus*, *Myopus schisticolor* and *Akodon azarae*) [3]. Many meiotic drivers are part of a complex gene drive system, due to a co-evolutionary arms race among distorters and their suppressors [1]. However, relatively little is known about how gene drive systems interact with ecological and environmental factors in natural populations and how such interactions contribute to their long-term stability [1].

The African buffalo (*Syncerus caffer*) of southern Africa may provide an opportunity to address these outstanding issues. A complex gene drive system involving sex-ratio distorters and suppressors was recently hypothesized for the buffalo population of Kruger National Park [6,7]. Further, male-deleterious alleles were observed in the same population, with which the gene drive system seems to interact [8]. Both the hypothesized gene drive system and the male-deleterious alleles were linked to environmental and pathogen stressors [8,9]. The gene drive system is unlikely to be restricted to one population alone. Here we study its possible presence and dynamics in the buffalo population of Hluhluwe-iMfolozi Park (HiP), a game reserve 280 km south of Kruger. In both populations bovine tuberculosis (BTB) is endemic, providing a valuable opportunity to evaluate short-term impacts on what was, until the 1800s, a contiguous population. However, the population in HiP is considerably smaller than the population in Kruger (4,000 individuals in a 900 km^2^ area *vs*. 37,000 individuals in a 20,000 km^2^ area), receives 50% more rain (annual rainfall 1979–2004: 804 mm *vs*. 537 mm), and has a markedly different BTB disease management strategy in place, *viz*., a yearly test-and-cull policy in which BTB-infected animals are removed from the population [9,10].

Two microsatellite studies in the Kruger buffalo have shown the occurrence of two types of male-deleterious alleles or multiple-allele haplotypes. These allele types (or haplotype variants) cause a negative effect in males on body condition and resistance to bovine tuberculosis (BTB) [8,9]. One group of microsatellite alleles was associated with a negative effect on the body condition of both sexes. This group was characterized by one deleterious-effect (DE) associated microsatellite allele at each of eight DE microsatellite loci. The other group of microsatellite alleles was associated with a negative effect on the body condition and health (higher BTB susceptibility) in males, but a positive effect in females. This group was characterized by various sexually-antagonistic-effect (SAE) associated microsatellite alleles at each of nine SAE microsatellite loci. The sexually antagonistic associations indicate that the SAE microsatellite alleles are linked to sexually antagonistic alleles or, alternatively, to haplotypes of closely linked male-deleterious and female-beneficial alleles.

Further, microsatellite alleles were mainly associated with deleterious traits in animals born after relatively wet 3yr-pre-birth-rainfall periods (‘genetic-measure by 3yr-pre-birth-rainfall’ interactions in logistic regression analysis with binary trait as dependent variable) [9]. The association with pre-birth rainfall suggests that the activity of the male-deleterious alleles is epigenetically influenced by parental body condition, considering that rainfall has a major effect on food resource availability [11]. This parental influence is not necessarily heritable (transgenerational) and may constitute a non-heritable (within generational) maternal effect, occurring in utero or shortly after birth [12,13]. The male-deleterious alleles probably occur genome-wide and at high frequencies, considering that only 17 microsatellite loci were used.

**Fig 1:**
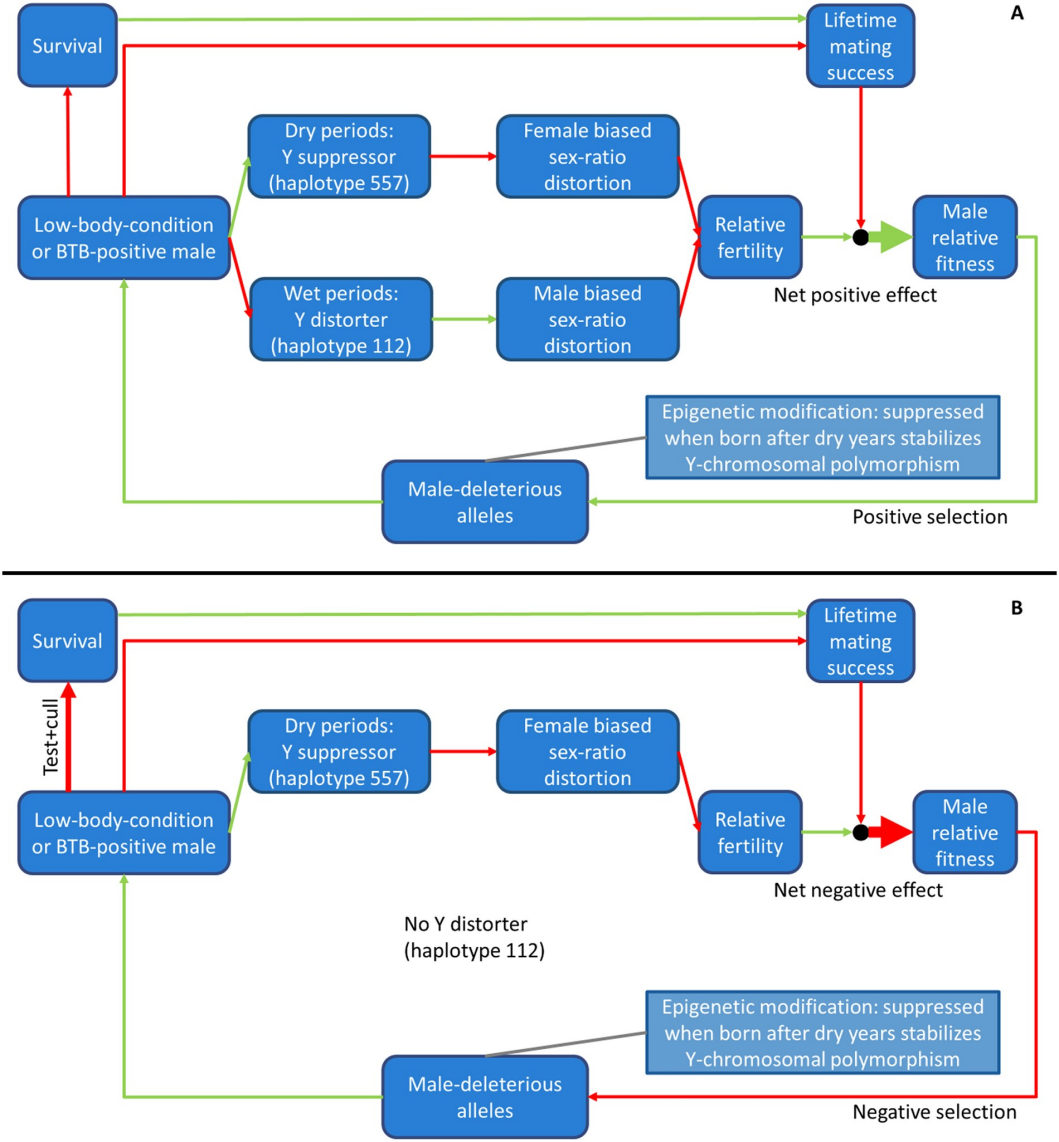
Mechanism underlying sex-ratio distortion and selection of male-deleterious alleles in Kruger (A) and HiP (B). Green lines: positive effects, red lines: negative effects. A) This mechanism has been described earlier, although not graphically [9]. Low body condition or BTB infection in males activates the Y-haplotype-557 linked Y suppressor during dry periods but inactivates the Y-haplotype-112 linked Y distorter during wet periods. Y-suppressor activation suppresses female-biased primary sex ratio distortion, while Y-distorter inactivation suppresses male-biased primary sex ratio distortion. Both processes diminish distortion-associated fertility loss. This increases the relative fertility of Y-distorter and Y-suppressor carriers. Low body condition or BTB infection decreases lifetime mating success directly, and indirectly through decreased survival. Increased relative fertility and decreased lifetime mating success have a net positive effect on male relative fitness. This results in positive selection and higher frequencies of male-deleterious alleles when the net relative fitness effect across sexes is positive. Higher male-deleterious allele frequencies negatively affect average male body condition and BTB resistance. This mechanism represents a positive feedback loop. Epigenetic suppression of male-deleterious alleles among animals born after 3yr-dry periods is hypothesized to result in negative frequency-dependent selection of the Y distorter-suppressor pair. This type of selection stabilizes Y-chromosomal polymorphism. B) Absence of a Y distorter in HiP is hypothesized to diminish the net positive effect of sex-ratio distortion on male relative fertility. The test-and-cull program is expected to increase the negative effect of male-deleterious alleles on male lifetime mating success, as males carrying these alleles have a relatively high chance of being infected with BTB and thus being culled. Decreased lifetime mating success and a smaller increase in relative fertility are hypothesized to have a net negative effect on male relative fitness. A lower male relative fitness results in negative selection and lower male-deleterious allele frequencies. This subsequently has a positive effect on average male BTB resistance. This mechanism represents a negative feedback loop.

The high frequencies of the male-deleterious alleles have been attributed to interactions with a Y-chromosomal sex-ratio distorter-suppressor pair, whose activity also seems to be influenced by body condition and health status [7–9]. The sex-ratio distorter-suppressor pair is linked to microsatellite-derived haplotypes; respectively Y-haplotype 112 and Y-haplotype 557 [6]. The underlying mechanism, which was earlier hypothesized in Van Hooft et al. 2018 [9], is explained in Table 1 and Fig 1A. The hypothesized mechanism consists of four components. 1) Among males with many male-deleterious alleles, low lifetime-mating success, due to low body condition and poor health, is offset by high relative fertility. 2) This offset is related to a negative effect of sex-ratio distorters on male fertility (a negative fertility effect is generally observed in species with sex-ratio distorters [3,7,14]). 3) These distorters are mainly active in males with few active male-deleterious alleles. 4) Epigenetic suppression of male-deleterious alleles among individuals born after 3yr-pre-birth dry conditions was hypothesized to stabilize Y-chromosomal polymorphism [9].

**Table 1:** Earlier findings that support the hypothesis that positive selection of male-deleterious alleles is driven by sex chromosomal meiotic drive. The deduction in this table has been presented in previous studies [7–9].

	Y-haplotype 557 dynamics	Y-haplotype 112 dynamics
1	Y-haplotype 557 is most frequent in cohorts born under dry circumstances [6,7].	Y-haplotype 112 is most frequent in cohorts born under wet circumstances [6,7].
2	Under dry circumstances, foetus sex ratio is female-biased [7].	Under wet circumstances, foetus sex ratio is male-biased [7].
3	From points 1 and 2 we deduce that Y-haplotype 557 increases in frequency when sex ratio becomes more female-biased	From points 1 and 2 we deduce that Y-haplotype 112 increases in frequency when sex ratio becomes more male-biased.
4	Point 3 suggests that that Y-haplotype 557 is linked to a Y suppressor. This Y suppressor is likely responding to an active X distorter which causes a female-biased sex ratio.	Point 3 suggests that Y-haplotype 112 is linked to a Y distorter. Males with a Y distorter produce an above average number of sons.
5	Sex-ratio distorters have a negative effect on male fertility [3,7,14]. Suppression of the X distorter prevents fertility loss. Thus, males with an active X distorter probably have a lower fertility than males with a suppressed X distorter.	Sex-ratio distorters have a negative effect on male fertility [3,7,14]. In case of Y distorters, this is due to a negative effect on the number of female offspring.
6	Food scarcity during dry circumstances has a negative effect on body condition and health [11].	Food abundance during wet circumstances has a positive effect on body condition and health [11].
7	Points 1, 2 and 6 suggest that the X distorter and Y suppressor are mainly active when body condition and health of their male carrier are low.	Points 1 and 6 suggest that the Y distorter is mainly active when body condition and health of its male carrier are high.
8	Male-deleterious alleles have a negative effect on male body condition and health [8,9].	
9	Points 7 and 8 suggest that male-deleterious alleles activate the Y suppressor.	Points 7 and 8 suggest that male-deleterious alleles suppress the Y distorter.
10	By activating the Y suppressor, male-deleterious alleles indirectly prevent fertility loss (step 5).	By suppressing the Y distorter, male-deleterious alleles prevent fertility loss (step 5).
11	Low body condition and health have a negative effect on fitness by lowering survival and mating success [15,16].	
12	Positive selection of male-deleterious alleles occurs when: Positive effect on relative male fitness (by preventing fertility loss, point 10) is larger than negative effect on male fitness by lowering body condition and health (point 11). Net positive fitness effect in males is larger than negative fitness effect in females, when averaged across all males and females.	

Despite the complexity of the underlying mechanism and its dependency on multiple hypothesises, we think it constitutes the most plausible explanation for a wide range of observations (S1 Text). In particular, the mechanism can explain stable Y-chromosomal polymorphism despite large year-to-year and season-to-season frequency fluctuations in Y-haplotypes [7]. Further, it can explain why during droughts males with many male-deleterious alleles have the highest reproductive success [8,9].

The male-deleterious alleles were expected to have a negative effect on population viability in Kruger by decreasing male body condition and by increasing disease occurrence; the latter directly in males and indirectly in females by contacting infected males [9]. The male-deleterious alleles may also occur in Hluhluwe-iMfolozi Park (HiP). However, we found that the frequencies of the deleterious-trait associated microsatellite alleles (i.e, DE and SAE alleles) were relatively low in Hluhluwe-iMfolozi Park (HiP) compared to Kruger (average frequency in HiP = 0.55 versus Kruger = 0.65; raw data from 12 microsatellites in [9,17]).

By way of background information, it is worth noting that the HiP buffalo population has the lowest genetic diversity (autosomal DNA, Y-chromosomal DNA and mitochondrial DNA) of any buffalo population studied so far [6,17–19], except for the small South African populations of St. Lucia and Addo (< 200 animals) [20]. Low genetic diversity can probably largely be attributed to a founder event, which is supported by microsatellite-based bottleneck tests [21]. The HiP buffalo population has been completely isolated for about 100 years and was founded by only a few individuals in 1895, increasing to 75 at most in 1929 [17]. Population size remained below 800 until the mid-1950s, due to wildlife culling in the 1930s and 1940s aimed at eradicating trypanosomiasis [21,22].

Random genetic drift caused by the founder event may by itself explain the relatively low frequencies of the DE and SAE alleles in HiP. However, low frequencies may also be caused by negative selection of male-deleterious alleles as Y-haplotype 112 was not observed among 180 HiP males typed previously (frequency ≤ 0.017, *P* = 0.05 [6,17]). In Kruger, this Y-haplotype was hypothesized to be linked a Y distorter [7]. Further, this Y distorter was hypothesized to contribute to net positive selection of male-deleterious alleles by its positive effect on male relative fertility (Table 1, Fig 1A) [9]. As a result, the negative effect of male-deleterious alleles on male lifetime mating success may result in net negative selection (Fig 1B). The possibility of negative selection on male-deleterious alleles forms the crux of this study. In contrast to Y-haplotype 112, Y-haplotype 557 is present in HiP (frequency = 0.33, 95% CI: 0.27, 0.41) [6,17]. In Kruger, this Y-haplotype was hypothesised to be linked to a Y suppressor (Table 1) [7].

Disease-focussed culling may contribute to negative selection by decreasing lifetime mating success of BTB-positive males (Fig 1B). BTB was first diagnosed in HiP in 1986 [23], and BTB prevalence was around 17% between 1999 and 2006 [10]. A test-and-cull program was initiated in HiP in 1999 (still ongoing in 2006) in an attempt to eradicate BTB. Buffalo were tested for BTB with only BTB-negative individuals released back into the park [21,24]. The culling was extensive, with 260–950 animals per year tested in a population of no more than 4000 individuals [10,21]. Four to six years of extensive disease-focussed culling (1999–2004; the samples in this study are from 2002–2004) may have provided additional negative selection pressure on male-deleterious alleles. The possibility of selection in HiP buffalo due to disease-focussed culling is supported by a recent genetic study [21]. This study found signatures of selection at the *INFG* gene and significant differences in number and frequency of rare alleles at closely linked microsatellites between BTB-positive and BTB-negative buffalo.

Here we report on the extent to which male-deleterious alleles are active in HiP buffalo. We assess whether these alleles are under negative selection and, if so, to what extent negative selection can be attributed to BTB, facilitated by the absence of an active Y distorter. More specifically we addressed the following four research questions.

1. Are DE and SAE allele frequency differences between HiP and Kruger correlated with genotype-phenotype association strength in Kruger (i.e., associations between microsatellite alleles and deleterious traits; here BTB infection and low body condition)? We hypothesized decreasing DE and SAE allele frequencies in HiP compared to Kruger with increasing association strength in Kruger. This would indicate that selection on the linked male-deleterious alleles is negative in HiP. Additionally, we tested for an influence of parental body condition on selection by correlating birth-year cohort allele frequencies with 3yr-pre-birth rainfall in HiP.
2. Does pre-birth rainfall have an influence on genotype-phenotype association strength in HiP (deleterious trait; here BTB infection)? We hypothesized the same statistical interaction between 3yr-pre-birth rainfall and genotype-phenotype association strength as in Kruger: i.e., no or weak association among individuals born after three dry years, but a strong positive association among individuals born after three wet years, consistent with epigenetic modification [9].
3. Is the frequency of Y-haplotype 557 (linked to a Y suppressor in Kruger) among male birth-year cohorts correlated with 3yr-pre-birth rainfall? We hypothesized a negative correlation, as observed in Kruger [6,7], which would indicate that the same sex-ratio suppressor is active in both populations.
4. Does female-biased primary sex-ratio distortion occur in HiP? In Kruger, both male- and female-biased sex-ratio distortion occurred among foetuses, which were attributed to respectively a Y distorter and a postulated X distorter [7,9]. All else being equal, one would expect a female-biased primary sex-ratio distortion in HiP if the Y distorter (linked to Y-haplotype 112) is absent. In particular, we hypothesized that birth-year cohort-specific sex ratios become more female-biased with decreasing 3yr-pre-birth rainfall, as was observed in Kruger with respect to foetus sex ratio [7]. This would indicate a maternal effect, which would be difficult to reconcile with post-birth differences in male and female mortality.

## Materials and methods

### Ethics

Ethical approval was not considered necessary, because BTB diagnosis and blood sample collection were conducted under the directive of the provincial parks authority, KwaZulu-Natal Wildlife, for other purposes. Thus the BTB test results and the use of blood samples in the present study are incidental. Full details of collection and sampling methods have been published before [10]. Briefly, buffalo were guided into bomas and subsequently immobilized according to standard operating procedures developed by KwaZulu-Natal Wildlife’s game capture veterinarians. Buffalo remained immobilized for 10–30 min during which time a blood sample was drawn and a BTB skin test administered. BTB-positive animals were slaughtered.

### Description of population and samples

The 960 km^2^ (28°S and 31–32°E) Hluhluwe-iMfolozi Park (HiP, South Africa) contains around 4000 African buffalo. Between 2002–2004, blood samples of 387 individual buffalo (158 males, 119 females) were genotyped from 13 breeding herds across all management regions of the park: Manzibomvu (two herds, Sep.-Oct. 2004), Nqumeni (one herd, Sep. 2002), Masinda (five herds, May-Sep. 2002, April 2003), Makhamasi (two herds, Apr. 2003) and Mbhuzane (three herds, May 2002) (S1 Fig). BTB testing using the single comparative intradermal tuberculin test (SCITT) and dental age estimation (to within approximately one year) were conducted, according to earlier described methods [22], on all except six of the genotyped individuals. No discernible differences were detected between the age-sex composition of captured buffalo and undisturbed groups [22]. Fourteen unlabelled samples from 2002–2004 were also genotyped (i.e., 401 genotyped buffalo in total). A further 553 individuals with known sex and age from the 2002–2004 roundups were used for logistic regression analysis of sex ratio in relation to 3yr-pre-birth rainfall and age.

### Molecular analyses

The same seventeen autosomal microsatellite loci as in two earlier Kruger buffalo genetics studies [8,9] were analysed using three core multiplex PCRs [25]. Only 1.5% (105/6817) of the PCR amplifications of the HiP samples were unsuccessful. The samples from Kruger and HiP were genotyped at the same time, using the same DNA sequencer. Therefore, alleles of the same length could be considered homologous. The microsatellite data from Kruger are available at the Dryad Digital Repository (doi:10.5061/dryad.s4cc3) [9]. Y-chromosomal microsatellite data from 157 HiP males were sourced from Van Hooft et al. 2007 [6].

In Kruger, eight autosomal microsatellite loci contained a majority allele (each with frequency > 0.63; *BM3517*, *BM4028*, *ETH010*, *ETH225*, *INRA006*, *INRA128*, *TGLA227* and *TGLA263*). Their average frequency per individual (i.e., average of eight alleles) was associated with low body condition, thereby indicating linkage to genes expressing a deleterious allele [8,9]. At the other nine autosomal microsatellite loci, the three most frequent alleles at each locus in Kruger were pooled. The average frequency per individual of these pooled alleles (i.e., average of nine pooled-threesomes) was associated with low body condition and increased BTB infection risk in males (*BM1824*, *BM3205*, *BM0719*, *CSSM019*, *DIK020*, *ILSTS026*, *SPS115*, *TGLA057* and *TGLA159*). These pooled alleles were also associated with high body condition and decreased BTB infection risk in females, thereby indicating linkage to genes expressing a sexually antagonistic allele [8,9]. Alternatively, the pooled alleles could be linked to haplotypes of closely linked male-deleterious and female-beneficial alleles. Identical to Van Hooft et al. 2018 [9], we refer to the former eight alleles as the deleterious-effect-associated alleles (DE_majority_ alleles) at the DE loci, and to the nine pooled-threesomes as the sexually-antagonistic-effect associated alleles (SAE_pooled_ alleles) at the SAE loci (S1 Table).

Wright’s *F*-statistics (no pooling of alleles), calculated with FSTAT 2.9.3.2, indicate little population structure in HiP (*F*_ST_ = 0.015, 95% CI: [+0.010, +0.020], *F*_IS_ = -0.015, 95% CI: [-0.040, +0.008], *F*_IT_ = 0.000, 95% CI: [-0.026, +0.025]). Further, median pairwise opposite-sex relatedness *r* (Lynch and Ritland 1999 [26], estimated with Genalex 6.5) among adults (≥ 5 years) within herds was low (*r* = 0.0057, with only 66 out of 544 pairs ≥ 0.2). As in Kruger, linkage disequilibrium (LD, calculated with FSTAT 2.9.3.2) was observed in HiP between locus pairs *CSSM019-BM1824*, *CSSM019-BM3205*, *BM1824-BM3205* and *INRA006-ILSTS026* (*P* < 0.00037 for each pair, Bonferroni-corrected α-level = 0.00037, α = 0.05). LD was probably due to physical linkage [8].

### Rainfall data

Annual rainfall data, with years running from October to September, were obtained from eight rainfall stations throughout HiP. These are updated rainfall data compared to an earlier genetic study on HiP buffalo [6], after discovery of inaccuracies. The period October-September was chosen because the wet season runs from October until March (S2 Fig). The mean annual rainfall in the period 1979–2004 was 804 mm (S2 and S3 Figs). Mean annual rainfall in the three years before the year of birth was used as an estimate of 3yr-pre-birth rainfall. In Kruger the following statistical effects were previously shown to be strongest with this 3yr-pre-birth-event period [6–9]: 1) correlations with Y-chromosomal haplotype frequencies, 2) correlations with expected heterozygosity at autosomal microsatellite loci, and 3) interactions with male-deleterious genetic effects in logistic regression analysis.

### Statistical analyses

#### Selection in relation to association strength with BTB and body condition

We analysed selection at the level of individual microsatellite alleles using non-parametric randomization tests. A randomization approach was chosen, because observations based on multiple alleles per locus and from different loci in LD cannot be assumed to be independent from one another. We randomized multilocus microsatellite genotypes (i.e., not on a locus-by-locus basis, every individual in the randomized datasets kept its original genotype), which guaranteed the same statistical dependence among alleles in the randomized data sets as in the observed data. We included only those alleles observed ≥ 15 times in southern Kruger (frequency ≈ 0.05) to reduce the number of statistical outliers.

For each individual allele in southern Kruger, where the strongest male-deleterious effects were observed [9], we estimated the association strength with BTB and body condition by comparing allele frequencies between low-body-condition BTB-positive individuals (*N*_males_ = 35, *N*_females_ = 37) and high-body-condition BTB-negative individuals (*N*_males_ = 39, *N*_females_ = 37). We compared low-body-condition BTB-positive and high-body-condition BTB-negative individuals from southern Kruger, because these two groups were characterized by considerably larger allele frequency differences than pairs of groups based on differences in body-condition status (low body condition *vs*. high body condition) alone or disease status (BTB-positive *vs*. BTB-negative) alone [9]. We therefore considered this comparison to be the most reliable indicator of association strength with BTB and body condition.

We denoted the sex-independent and sexually-antagonistic association strength of microsatellite alleles by the ratios *A*_sex-indep_ and *A*_sex-anta_ respectively. In estimating these ratios, we define with respect to individual allele frequency *a* in males (subscript *m*) or females (subscript *f*) that are in high (subscript *h*) or low (subscript *l*) body condition and are BTB-positive (+) or BTB-negative (-) using the notation *a*_sex,condition,BTB state_. Thus, for example, *a*_*f*,*h*,-_ represents the frequency of the allele in question in high-body-condition BTB-negative females, while *a*_*m*,*l*,+_ represents the frequency of the allele in question in low-body-condition, BTB-positive males. From this it follows that

$$A_{sex-indep}=\frac{a_{m,l,+}+a_{f,l,+}}{a_{m,h,-}+a_{f,h,-}}$$ (1)

and

$$A_{sex-anta}=\frac{a_{m,l,+}+a_{f,h,-}}{a_{m,h,-}+a_{f,l,+}}$$ (2)

To analyse whether effects of *A*_sex-indep_ could be attributed to one of the two sexes we also estimated the sex-specific association strength, denoted by the ratios *A*_male-spec_ and *A*_female-spec_, as:

$$A_{male-spec}=\frac{a_{m,l,+}}{a_{m,h,-}}$$ (3)

and

$$A_{female-spec}=\frac{a_{f,l,+}}{a_{f,h,-}}$$ (4)

We note *A* values > 1 indicate association with deleterious effects (male-deleterious except *A*_female-spec_ which relates to female-deleterious effects), while *A* values < 1 indicate association with beneficial effects. Larger deviations from 1 indicate stronger associations. We only included the individual DE alleles with *A*_male-spec_ and *A*_female-spec_ either both > 1 or both < 1 (DE_indv_ alleles, including 7 out of 8 DE_majority_ alleles, excluding the DE_majority_ allele at *INRA006* because *A*_female-spec_ was < 1; 16 alleles in southern Kruger and 14 alleles in HiP; S2 Table). Further, we only included the individual SAE alleles (SAE_indv_ alleles) with either *A*_male-spec_ > 1 *and A*_female-spec_ < 1 or *vice versa* (individual opposite-sex difference alleles, SAE_indvO_ alleles; 31 alleles in southern Kruger and 20 alleles in HiP; S3 and S4 Tables). DE_indv_ alleles and SAE_indvO_ alleles were tested in relation to *A*_sex-indep_ and *A*_sex-anta_, respectively.

As a negative control, we also analysed the SAE_indv_ alleles with *A*_male-spec_ and *A*_female-spec_ either both > 1 or both < 1 (individual no-sex difference alleles, SAE_indvN_ alleles; 20 alleles in southern Kruger and 14 alleles in HiP; S3 and S4 Tables). These SAE_indvN_ alleles were tested in relation to *A*_sex-indep_. We did not expect to see signatures of selection with the SAE_indvN_ alleles, because, considering their sex-independence, they are probably only weakly associated with sexually antagonistic effects. No negative control was possible with the DE_indv_ alleles because of the low number of alleles with opposite-sex differences (five alleles in southern Kruger and four alleles in HiP).

We performed two tests to see whether allele frequency differences between HiP and Kruger, estimated as the ratio of allele frequency in HiP to allele frequency in northern Kruger (HiP/northern-Kruger frequency ratio, HiP: *N*_individuals_ = 401, northern Kruger: *N*_individuals_ = 138), were associated with *A*_sex-indep_ and *A*_sex-anta_. A negative association with *A*_sex-indep_ in case of DE_indv_ alleles and with *A*_sex-anta_ in case of SAE_indvO_ alleles would be indicative of negative selection in HiP (research question 1). Allele frequencies from northern Kruger, rather than southern Kruger, were used to estimate the HiP/northern-Kruger frequency ratio in order to make this variable as independent as possible from *A*_sex-indep_ and *A*_sex-anta_. We additionally explored associations between the HiP/northern-Kruger frequency ratio of SAE_indvN_ alleles on the one hand and *A*_male-spec_ and *A*_female-spec_ on the other (negative control analysis).

In the first test (Test 2 in Table 2), we assessed whether *A*_sex-anta_ (SAE_indvO_ alleles) and *A*_sex-indep_ (SAE_indvN_ alleles) significantly differed between alleles observed in both populations and alleles observed only in Kruger. This test could only be conducted for the SAE_indvO_ and SAE_indvN_ alleles, because only two DE_indv_ alleles were unique to Kruger. In the second test (Test 3 in Table 2), which was performed on alleles observed in both populations, we assessed whether the HiP/northern-Kruger frequency ratio was significantly correlated with *A*_sex-indep_ and *A*_sex-anta_.

**Table 2:** Overview of the conducted statistical tests and interpretation of results. *A*: association strength with BTB and body condition in southern Kruger, MDL_*s*_: standardized male-deleterious load, PBR: 3yr-pre-birth rainfall, DE_majority_ and SAE_pooled_ alleles: microsatellite allele types defined according to Van Hooft et al. 2018 [9], DE_indv_, SAE_indvO_ and SAE_indvN_ alleles: newly defined microsatellite allele types, see also Table 3. Test^a^: addressed research question is mentioned between brackets.

Test^a^	Independent variables	Dependent response	Probability	Interpretation
1 (1)	DE_majority_ and SAE_pooled_ alleles	Kruger-HiP allele freq. difference	*P* = 0.19	Weak support for negative selection of male-deleterious alleles
2 (1)	SAE_indvO_ and SAE_indvN_ alleles	Rank difference in *A* between observed and non-observed alleles	*P* = 0.0026	Negative selection of male-deleterious alleles
3 (1)	DE_indv_ alleles	Correlation between *A* and HiP/northern-Kruger freq. ratio	*P* = 0.32	Weak support for negative selection of male-deleterious alleles
3 (1)	SAE_indvO_ and SAE_indvN_ alleles	Correlation between *A* and HiP/northern-Kruger freq. ratio	*P* = 0.00021	Negative selection of male-deleterious alleles. Newly identified allele type: male-specific deleterious alleles.
4	DE_indv_ alleles	Correlation between birth-year cohort freq. and PBR	*P* > 0.4	No significance
4	SAE_indvO_ and SAE_indvN_ alleles	Correlation between birth-year cohort freq. and PBR	*P* = 0.00018	Parental body condition affects selection of male-deleterious alleles. Newly identified allele type: male-specific beneficial alleles.
5 (2)	MDL_*s*_ and SAE_indvN,*A*<1_ alleles	MDL_*s*_-PBR interaction and total freq.-PBR interaction in logistic regression	*P* = 0.0060	Epigenetic suppression of male-deleterious and male-beneficial allele activity relative to PBR
6 (3)	Y-haplotype 557	Correlation between birth-year cohort freq. and PBR	*P* = 0.043	Increasing Y-suppressor activity with decreasing PBR
7 (4)	Sex ratio	Logistic regression between sex and PBR	*P* = 0.0026	Sex-ratio becomes more female-biased with decreasing PBR

Additionally, we tested for selection by correlating birth-year cohort frequencies of DE_indv_ and SAE_indv_ alleles with 3yr-pre-birth rainfall (Test 4 in Table 2). We excluded birth-year cohorts with ≤ 3 individuals to minimize the number of outliers. Significant correlations would indicate parental body condition as a selective agent, as previously has been observed in Kruger [8]. In this test, we made a distinction between alleles with *A* > 1 and alleles with *A* < 1 (*A* = 1 was not observed), which resulted in six allele types: DE_indv,*A*>1_, DE_indv,*A*<1_, SAE_indvO,*A*>1_, SAE_indvO,*A*<1_, SAE_indvN,*A*>1_ and SAE_indvN,*A*<1_ alleles (Table 3).

**Table 3:** Individual microsatellite allele types tested for selection. DE_indv_: in relation to *A*_sex-indep_, SAE_indvO_: in relation to *A*_sex-anta_, SAE_indvN_: in relation to *A*_male-spec_.

Microsatellite allele type	Trait association in southern Kruger	Linked to allele type at coding genes
DE_indv,*A*>1_	♂ and ♀: low-body-condition BTB-pos.	Deleterious alleles
DE_indv,*A*<1_	♂ and ♀: high-body-condition BTB-neg.	No clear linkage
SAE_indvO,*A*>1_	♂: low-body-condition BTB-pos. ♀: high-body-condition BTB-neg.	Sexually antagonistic alleles or haplotypes of male-deleterious and female-beneficial alleles
SAE_indvO,*A*<1_	♂: high-body-condition BTB-neg. ♀: low-body-condition BTB-pos.	No clear linkage
SAE_indvN,*A*>1_	♂: low-body-condition BTB-pos.	Male-specific deleterious alleles
SAE_indvN,*A*<1_	♂: high-body-condition BTB-neg.	Male-specific beneficial alleles

Significance of Tests 2 and 3 was estimated by randomizing multilocus microsatellite genotypes from southern Kruger among groups of buffalo based on body-condition and disease status (low-body-condition BTB-positive males, high-body-condition BTB-negative males, low-body-condition BTB-positive females and high-body-condition BTB-negative females). Randomizations were not performed among individuals from different populations because LD strength between loci can vary among populations, resulting in different LD strengths in the randomized data sets as compared to the observed data. The classification as DE_indv_, SAE_indvO_, or SAE_indvN_ allele was kept the same as in the observed data. Significance of Test 4 was estimated by randomizing multilocus microsatellite genotypes from HiP among birth-year cohorts. Two-sided probabilities were estimated as twice the fraction of random data sets showing the same or larger statistical value than the original data set (same or smaller when original value was negative), using 100,000 randomizations implemented using Excel 2016. The statistical value was difference in average rank of *A* in Test 2 and Spearman *ρ* in Tests 3 and 4. In Test 4, the combined probability of various allele types was estimated using average |*ρ*| as the statistical value.

We performed three logistic regression analyses to study: 1) statistical interactions between individual genetic background and 3yr-pre-birth rainfall, 2) the effect of 3yr-pre-birth rainfall on Y-haplotype status and 3) the effect of age and 3yr-pre-birth rainfall on sex. The logistic regression analyses were conducted using the ‘lme4’ package (version 1.1.13) in R. Herd affiliation and sampling year were incorporated as random intercepts in a mixed modelling approach.

#### Statistical interactions between individual genetic background and 3yr-pre-birth rainfall

Identical to the methodology in Van Hooft et al. 2018 [9], male-deleterious load (MDL) was based on the average fraction of homozygous and heterozygous DE_majority_ and SAE_pooled_ alleles per individual: HomDE_majority_, HomSAE_pooled_, HetDE_majority_ and HetSAE_pooled_. Heterozygosity was not calculated as 1-homozygosity, but as the fraction of heterozygous alleles among the loci that were not homozygous for DE_majority_ or SAE_pooled_ alleles [9]. This approach made homozygosity and heterozygosity statistically independent from one another [9]. Using *m* and *f* subscripts to denote that quantities were estimated separately among the male and female subgroups, male-deleterious load (MDL) was estimated in males as:

$${MDL}_{m}={HomDE}_{majority,m}+0.5\times {HetDE}_{majority,m}+{HomSAE}_{pooled,m}+0.5\times {HetSAE}_{pooled,m}$$ (5)

and in females as:

$${MDL}_{f}={HomDE}_{majority,f}+0.5\times {HetDE}_{majority,f}-{HomSAE}_{pooled,f}-0.5\times {HetSAE}_{pooled,f}$$ (6)

MDL_*m*_ and MDL_*f*_ did not significantly deviate from normality (Shapiro-Wilk test using SPSS 23: *P* > 0.8). Further, we explored whether the average total per-locus frequency of the SAE_indvN,*A*<1_ alleles (i.e., after pooling of all alleles per locus, Table 3) showed interaction with 3yr-pre-birth rainfall in logistic regression analysis. SAE_indvN_ alleles were initially not expected to be linked to alleles at coding genes (the previously mentioned negative control). The SAE_indvN,*A*>1_ alleles (Table 3) were not considered for logistic regression analysis because they were relatively rare and to keep the number of events per predictor variable (EPV) above the recommended minimum of five [27]. To aid in model convergence, all continuous variables were standardized per sex by subtracting the mean of each variable from each observation and dividing the result by the standard deviation of that variable. To increase EPV, the standardized estimates of MDL_*m*_ and MDL_*f*_ were treated as one independent variable: standardized MDL (MDL_*s*_).

In the first regression model 3yr-pre-birth rainfall, MDL_*s*_ and their interaction were included as fixed independent variables with BTB-infection status as dependent variable (Eq 7). A significant interaction would indicate epigenetic modification (research question 2). In the second regression model, we also included the following fixed independent variables: SAE_indvN,*A*<1_ allele frequency (average total per-locus frequency of all SAE_indvN,*A*<1_ alleles), sex, three-way interaction between SAE_indvN,*A*<1_ allele frequency, sex and 3yr-pre-birth rainfall, and all lower order two-way interactions (Eq 8). The three-way interaction and the ‘SAE_indvN,*A*<1_ allele frequency by sex’ interaction were included to test for sex-specific differences. The other two-way interactions were included because of hierarchical completeness. EPV of the regression models was 5.2 (47 BTB-positive individuals and 9 predictor variables considered).

$$logit(p)=b_{0}+b_{1}{MDL}_{s}+b_{2}{PBR}_{s}+b_{3}{MDL}_{s}{PBR}_{s}$$ (7)

$$\begin{array}{l} \text{logit}\left(p\right)=\\ b_{0}+b_{1}{\text{MDL}}_{s}+b_{2}{\text{SAE}}_{\text{indvN},s}+b_{3}{\text{PBR}}_{s}+\\ b_{4}\text{sex}+b_{5}{\text{MDL}}_{s}{\text{PBR}}_{s}+b_{6}{\text{SAE}}_{\text{indvN},s}{\text{PBR}}_{s}+b_{7}{\text{SAE}}_{\text{indvN},s}\text{sex}+\\ b_{8}{\text{PBR}}_{s}\text{sex}+b_{9}{\text{SAE}}_{\text{indvN},s}{\text{PBR}}_{s}\text{sex} \end{array}$$ (8)

Where: *p* = probability of BTB infection, MDL_*s*_ = standardized MDL, PBR_*s*_ = standardized 3yr-pre-birth rainfall, SAE_indvN,*s*_ = standardized total per-locus frequency of the SAE_indvN,*A*<1_ alleles, sex = categorical variable (male = 1, female = 0).

#### Y-suppressor activity

We conducted a logistic regression between Y-haplotype status and 3yr-pre-birth rainfall (*N*_individuals_ with Y-haplotype 557 = 53, *N*_individuals_ with other Y-haplotypes = 104, EPV = 53; Eq 9). Non-significance of logistic regression could be due to violation of the assumption of linearity of independent variables and log odds. Therefore, we also analysed, using SPSS 23, whether there was a significant Spearman correlation between Y-haplotype 557 frequency per birth-year cohort and 3yr-pre-birth rainfall, weighted by sample size per birth-year cohort. Significance of either test would indicate that Y-haplotype 557 is linked to an active sex-ratio suppressor (research question 3). We think so, because in Kruger the association of this Y-haplotype with pre-birth rainfall was interpreted as being indicative of sex-ratio suppressor activity (Table 1, S1 Text) [7].

$$logit(p)=b_{0}+b_{1}PBR$$ (9)

Where: *p* = probability of carrying Y-haplotype 557, PBR = 3yr-pre-birth rainfall.

#### Sex-ratio distortion

We performed a logistic regression with sex as the dependent variable and age (in years, age ≤ 16 years; the maximum observed male age) and 3yr-pre-birth rainfall as the independent variables (*N*_males_ = 415; *N*_females_ = 502, EPV = 207.5; Eq 10). To aid in model convergence, 3yr-pre-birth rainfall and age were standardized by subtracting the mean of each variable from each observation and dividing the result by the standard deviation of that variable. The birth sex ratio (fraction males) per birth-year cohort was predicted by extrapolating to age = 0. We expected a negative effect (i.e., lower sex ratio) of age because of the relatively short male lifespan in most long-lived vertebrates [28], and a positive effect (i.e., higher sex ratio) of 3yr-pre-birth rainfall because of a hypothesized decreasing X-distorter activity with increasing 3yr-pre-birth rainfall [9]. Because of the absence (or very low frequency) of a Y distorter we expected most birth-year cohorts to have a female-biased birth sex ratio (research question 4).

$$logit(p)=b_{0}+b_{1}{age}_{s}+b_{2}{PBR}_{s}$$ (10)

Where: *p* = probability of being male, age_*s*_ = standardized age, PBR_*s*_ = standardized 3yr-pre-birth rainfall.

The raw data set supporting the results of this article can be found in the Dryad Digital Repository: XXX, http://doi.org/10.5061/dryad.xxx (http://datadryad.org).

## Results

Our results reveal a complex set of interconnected genetic factors. To assist the reader in making these connections, we have provided a table (Table 2), next to the figure of the hypothesized underlying mechanism in the Introduction section (Fig 1), on how the results link together.

### Allele frequency differences between HiP and northern Kruger in relation to genotype-phenotype association strength (research question 1)

DE_majority_ and SAE_pooled_ allele frequencies were on average lower in HiP than in northern Kruger, but non-significantly so (average frequency HiP = 0.54, Kruger = 0.66; Wilcoxon signed-rank test: *P* = 0.19, S2 Text, S4 Fig; Test 1 in Table 2). However, significant differences were observed with respect to the individual microsatellite alleles.

**Fig 2:**
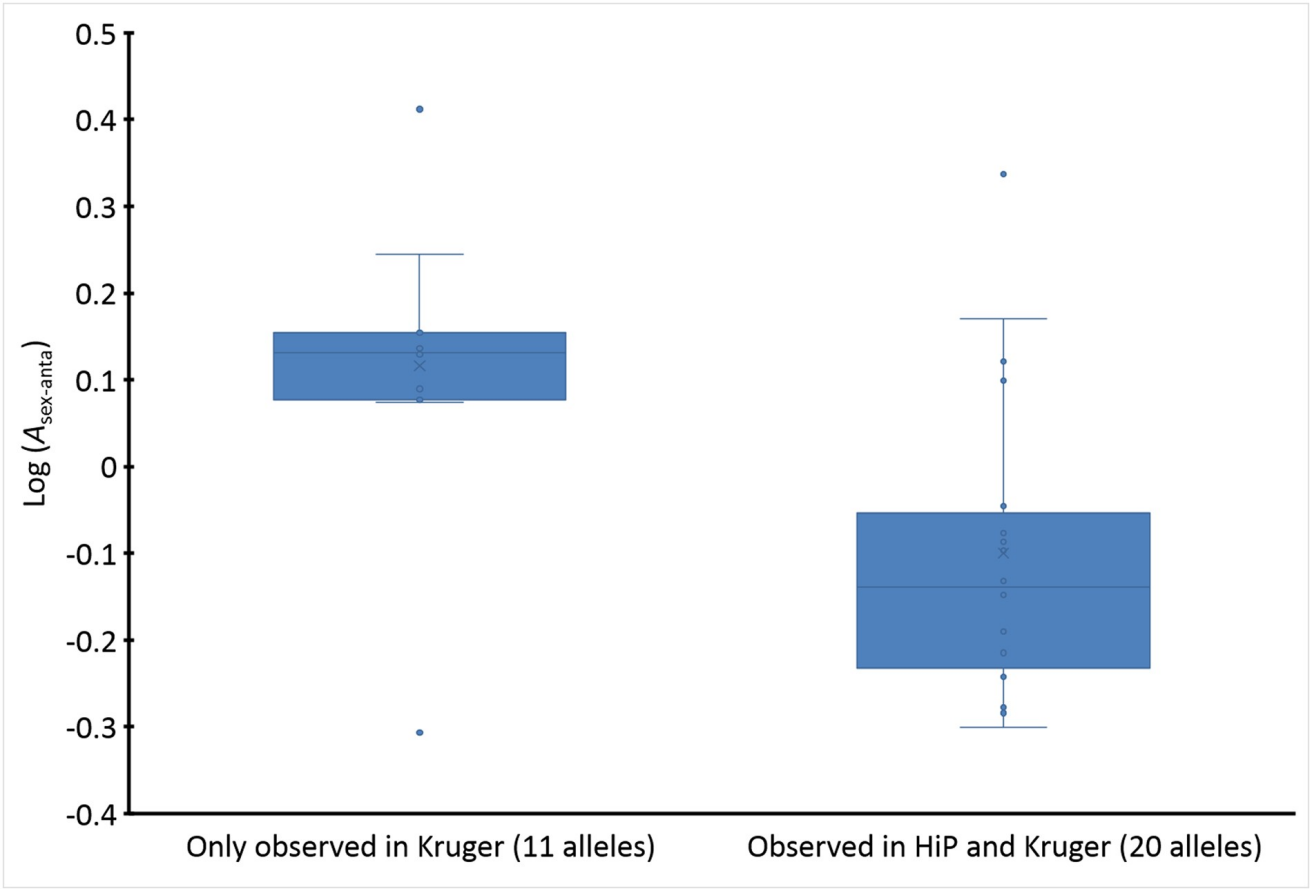
Difference in *A*_sex-anta_ between SAE_indvO_ alleles from Kruger observed and not observed in HIP. Median difference = 0.27, *P* = 0.0049. Test 2, Table 2.

The Kruger-specific SAE_indvO_ alleles (i.e., not observed in HiP) showed significantly larger *A*_sex-anta_ values than those also observed in HiP (*A*_sex-anta_: *P* = 0.0049; SAE_indvN_ alleles, *A*_sex-indep_: *P* = 0.76 (also larger values); Fig 2, Test 2 in Table 2). The results could not be attributed to allele frequency differences, because the average frequencies of SAE_indvO_ alleles in Kruger showed only small differences between those observed and not observed in HiP (average frequencies: observed alleles = 0.15, non-observed alleles = 0.16, unequal variance *t*-test: *P* = 0.75; S3 and S4 Tables).

**Fig 3:**
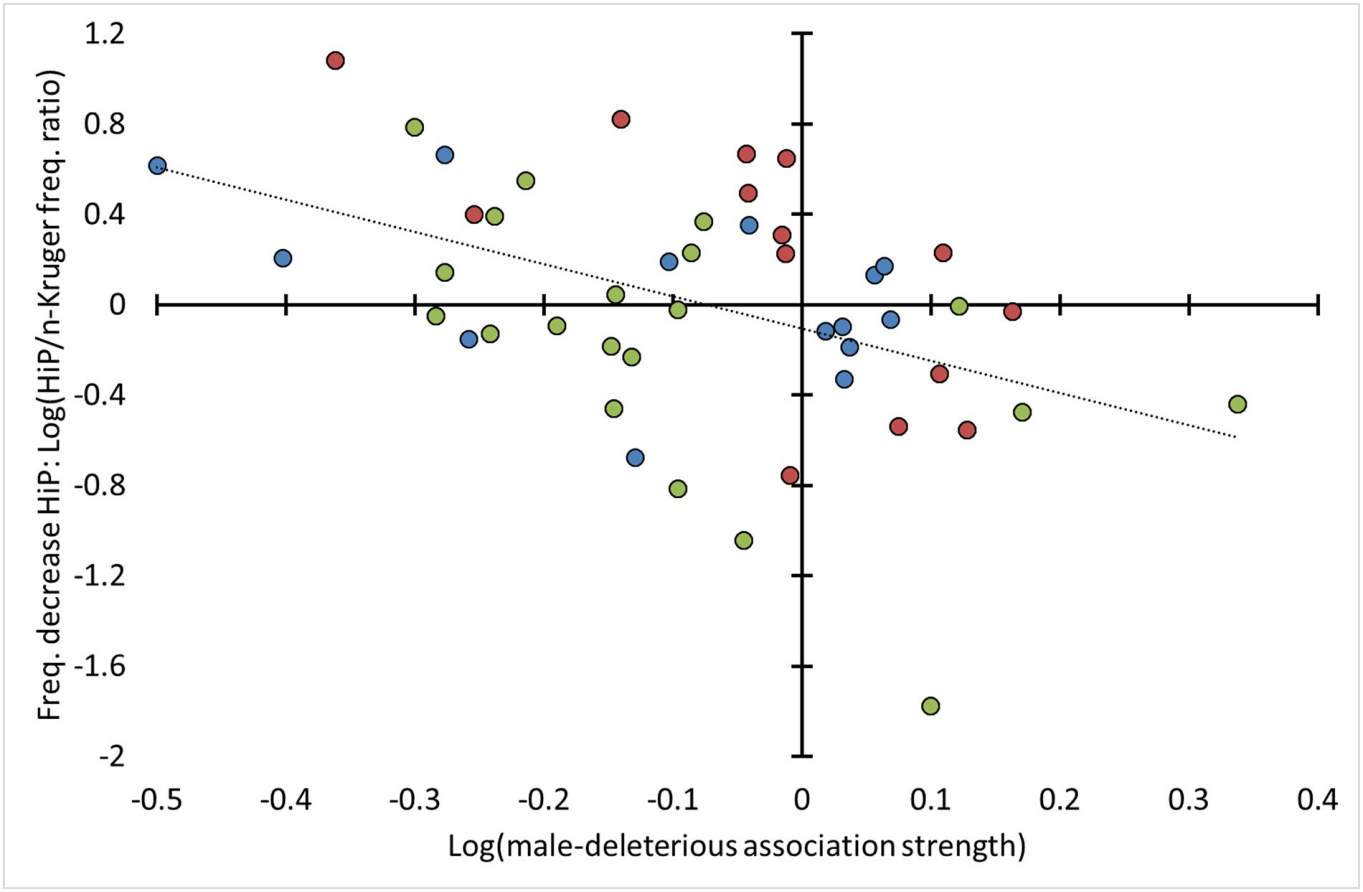
Negative correlations between allele frequency decrease in HiP and male-deleterious association strength. Blue data points: DE_indv_ alleles (*ρ* = -0.38, *P* = 0.32, *N*_alleles_ = 14; independent variable is *A*_sex-indep_), red data points: SAE_indvN_ alleles (*ρ* = -0.78, *P* = 0.0030, *N*_alleles_ = 14, independent variable is *A*_male-spec_), green data points: SAE_indvO_ alleles (*ρ* = -0.50, *P* = 0.023, *N*_alleles_ = 20; independent variable is *A*_sex-anta_), *N*_alleles_ = 48, combined *P* = 0.00033 (*Z*-transform test based on the three Spearman correlations). Test 3, Table 2.

Among the DE_indv_ alleles observed in HiP there was a non-significant negative correlation between *A*_sex-indep_ and the HiP/northern-Kruger frequency ratio (*ρ* = -0.38, *P* = 0.32; Fig 3, Test 3 in Table 2). However, among the SAE_indvO_ and SAE_indvN_ alleles observed in HiP, there was a significant negative correlation between respectively *A*_sex-anta_ and *A*_sex-indep_ on the one hand and the HiP/northern-Kruger frequency ratio on the other (SAE_indvO_, *A*_sex-anta_: *ρ* = -0.50, *P* = 0.023; SAE_indvN_, *A*_sex-indep_: *ρ* = -0.60, *P* = 0.040; Fig 3, Test 3 in Table 2).

The relatively large *A*_sex-anta_ values among Kruger-specific SAE_indvO_ alleles and the negative correlation between *A*_sex-anta_ and the HiP/northern-Kruger frequency ratio among SAE_indvO_ alleles also observed in HiP provide strong support for negative selection (*Z*-transform test: combined *P* = 0.00032). This selection occurred on the linked sexually antagonistic alleles or haplotypes of male-deleterious and female-beneficial alleles. The similar results with respect to the *A*_sex-indep_ values of the SAE_indvN_ alleles, the negative-control analyses, were not expected (large values when Kruger-specific and negative correlation with the HiP/northern-Kruger frequency ratio when also observed in HiP; *Z*-transform test: combined *P* = 0.095). Rather than considering these to be false positive results, they may indicate negative selection of another type of male-deleterious allele.

The frequency decrease of the SAE_indvN_ alleles in HiP was much more pronounced in relation to *A*_male-spec_ (Table 3). *A*_male-spec_ values were relatively large among Kruger-specific alleles and showed a negative correlation with the HiP/northern-Kruger frequency ratio among alleles also observed in HiP (rank difference Kruger-specific alleles *vs*. alleles also observed in HiP: *P* = 0.15; correlation with HiP/northern-Kruger frequency ratio: *ρ* = -0.78, *P* = 0.0030; *Z*-transform test: combined *P* = 0.0018; Fig 3 and S5 Fig; no significance with *A*_female-spec_: *P* ≥ 0.16). These results indicate linkage to alleles that are predominantly deleterious to males (male-specific deleterious alleles, Table 3). When considered together, SAE_indvO_ and SAE_indvN_ alleles showed a highly significant frequency decrease in HiP in relation to association strength (Z-transform tests; rank differences: combined *P* = 0.0026, correlations: combined *P* = 0.00021; Tests 2 and 3 in Table 2).

The SAE_indvO,*A*>1_ alleles (Table 3) showed a very strong frequency decrease in HiP relative to northern Kruger (average total frequency per locus: northern Kruger = 0.35, HiP = 0.06; S5 Fig). The SAE_indvN,*A*>1_ alleles (Table 3) on average halved in frequency in HiP relative to northern Kruger (average total frequency per locus: northern Kruger = 0.14, HiP = 0.07; S6 Fig).

### Effect of 3yr-pre-birth rainfall on allele frequencies

**Fig 4:**
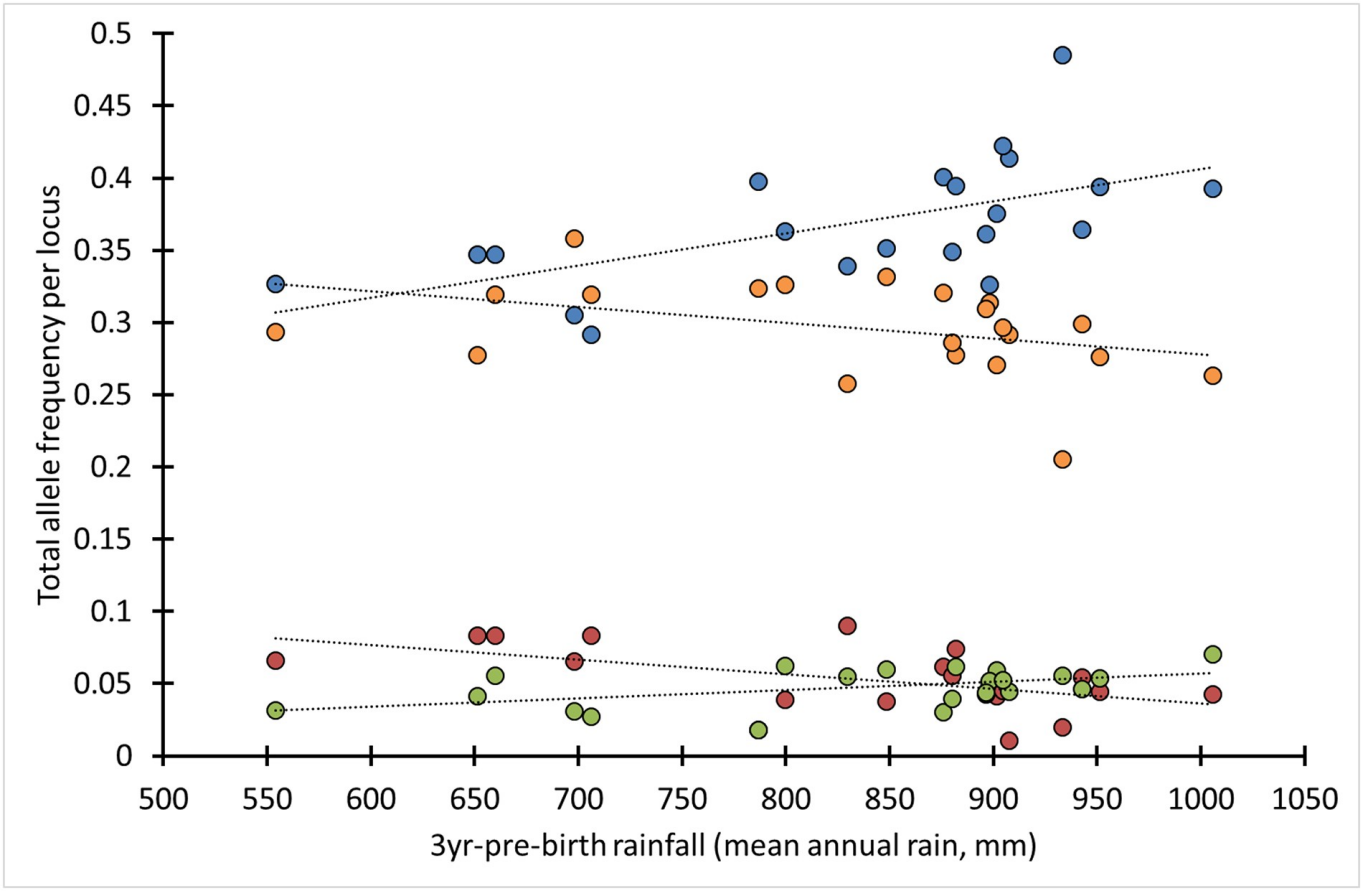
Correlations between birth-year cohort frequencies of SAE_indv_ alleles and 3yr-pre-birth rainfall. Blue data points: SAE_indvN,*A*<1_ alleles (nine alleles at eight loci): *ρ* = 0.62, *P* = 0.0051; red data points: SAE_indvN,*A*>1_ alleles (five alleles at five loci): *ρ* = -0.52, *P* = 0.017; green data points: SAE_indvO,*A*>1_ alleles (four alleles at four loci): *ρ* = 0.42, *P* = 0.083; orange data points: SAE_indvO,*A*<1_ alleles (16 alleles at nine loci): *ρ* = -0.49, *P* = 0.031; average |*ρ|* = 0.51, *P* = 0.00018; *N*_birth-year-cohorts_ = 21, ≥ 4 individuals per birth-year cohort genotyped, *N*_individuals_ = 380. SAE_indvO_ alleles based on *A*_sex-anta_, SAE_indvN_ alleles based on *A*_male-spec_. Test 4, Table 2.

Average total frequency per locus per birth-year cohort (i.e., alleles pooled per locus per birth-year cohort) of both the SAE_indvO_ (based on *A*_sex-anta_) and SAE_indvN_ (based on *A*_male-spec_) alleles, but not the DE_indv_ alleles (*P* > 0.4), correlated with 3yr-pre-birth rainfall. This observation indicates that selection of SAE_indv_ alleles was influenced by parental body condition (Fig 4, Test 4 in Table 2, Table 3). Correlation with 3yr-pre-birth rainfall was positive for SAE_indvO,*A*>1_ alleles (*ρ* = 0.42, *P* = 0.083) and SAE_indvN,*A*<1_ alleles (*ρ* = 0.62, *P* = 0.0051), and negative for SAE_indvO,*A*<1_ alleles (*ρ* = -0.49, *P* = 0.031) and SAE_indvN,*A*>1_ alleles (*ρ* = -0.52, *P* = 0.017; four correlations combined: average |*ρ*| = 0.51, *P* = 0.00018; Fig 4).

The correlations of the SAE_indvO,*A*<1_ and SAE_indvN,*A*<1_ alleles, originally hypothesized not to be linked to any particular protein-coding allele, could not entirely be an artefact of the correlations of the SAE_indvO,*A*>1_ and SAE_indvN,*A*>1_ alleles, as the total frequencies of the latter two groups combined did not show any meaningful correlation with 3yr-pre-birth rainfall (Pearson *r* = -0.21, *P* = 0.37). Thus, these correlations indicate linkage to at least three types of alleles or haplotypes (Table 3): sexually-antagonistic alleles or haplotypes of linked male-deleterious and female-beneficial alleles (linked to SAE_indvO,*A*>1_ alleles), male-specific deleterious alleles (linked to SAE_indvN,*A*>1_ alleles) and male-specific beneficial alleles (linked to SAE_indvN,*A*<1_ alleles). The last allele type is supported by an effect of 3yr-pre-birth rainfall on its association with BTB susceptibility (Research question 2, see next section). Although the SAE_indvO,*A*<1_ alleles may be linked to female-beneficial alleles, their negative correlation with pre-birth rainfall may also be an artefact of the significant positive correlation involving the SAE_indvN,*A*<1_ alleles.

### Effect of 3yr-pre-birth rainfall on genotype-phenotype association strength (research question 2)

Logistic regression analysis did not show a significant effect of the interaction between MDL_*s*_ and 3yr-pre-birth rainfall on BTB-infection risk when only MDL_*s*_ and 3yr-pre-birth rainfall were included as main factors (*P* = 0.16, AIC = 261.8). The interaction became near-significant (*P* = 0.068, AIC = 262.7; Table 4), when also the average total per-locus frequency of the SAE_indvN,*A*<1_ alleles (Table 3) was included as main factor, together with its interactions with 3yr-pre-birth rainfall and sex. The interaction indicates that MDL_*s*_ had the strongest effect on BTB-infection risk after relatively wet pre-birth years, as was earlier observed in Kruger [9].

**Table 4:** Logistic regression between BTB-infection risk (dependent) and MDL_*s*_, SAE_indvN,*A*<1_ allele frequency and 3yr-pre-birth rainfall. Males: *N*_BTB-positive_ = 17, *N*_BTB-negative_ = 141, females: *N*_BTB-positive_ = 30, *N*_BTB-negative_ = 194, EPV = 5.2, Pearson correlation between main factors: |*r*| ≤ 0.22. Test 5, Table 2.

Parameter	Mean (logit scale)	SE	Probability
MDL_*s*_ (standardized; *x*_1_)	0.002	0.184	0.992
SAE_indvN,*A*<1_ (standardized; *x*_2_)	0.169	0.224	0.450
Pre-birth rainfall (standardized; *x*_3_)	-0.386	0.209	0.065
Sex (♂ = 1, ♀ = 0; *x*_4_)	-0.800	0.422	0.058
*x*_1_-by-*x*_3_ interaction	0.334	0.183	0.068
*x*_2_-by-*x*_3_ interaction	-0.019	0.270	0.945
*x*_2_-by-*x*_4_ interaction	-0.868	0.407	0.033
*x*_3_-by-*x*_4_ interaction	0.875	0.393	0.026
*x*_2_-by-*x*_3_-by-*x*_4_ interaction	1.183	0.500	0.018
Intercept	-2.317	0.878	0.008

The two- and three-way interactions involving the male-beneficial-linked SAE_indvN,*A*<1_ alleles (Table 3) indicate that these alleles were negatively associated with BTB-infection risk, but only in males (*x*_2_-by-*x*_4_ interaction: Table 4, *P* = 0.033) and mainly after relatively dry pre-birth years (*x*_2_-by-*x*_3_-by-*x*_4_ interaction: *P* = 0.018, Table 4, S5 and S6 Tables). This is opposite to what was observed with MDL_*s*_, namely a positive association after relatively wet pre-birth years. The occurrence of two ‘genetic-measure by rainfall’ interactions was highly significant (*Z*-transform test: *P* = 0.0060, combined probability of the *x*_2_-by-*x*_3_-by-*x*_4_ and *x*_1_-by-*x*_3_ interactions, Test 5 in Table 2). We observed no meaningful interactions (*x*_1_-by-*x*_3_ in both models, *x*_2_-by-*x*_3_-by-*x*_4_: *P* ≥ 0.16) when, as a control, we replaced 3yr-pre-birth rainfall with age or 3yr-post-birth rainfall (0–2 years after birth, 1–2 year post-birth periods in case of 0–1 year olds) in the regression equations.

### Effect of 3yr-pre-birth rainfall on Y-haplotype-557 birth-year cohort frequency (research question 3)

**Fig 5:**
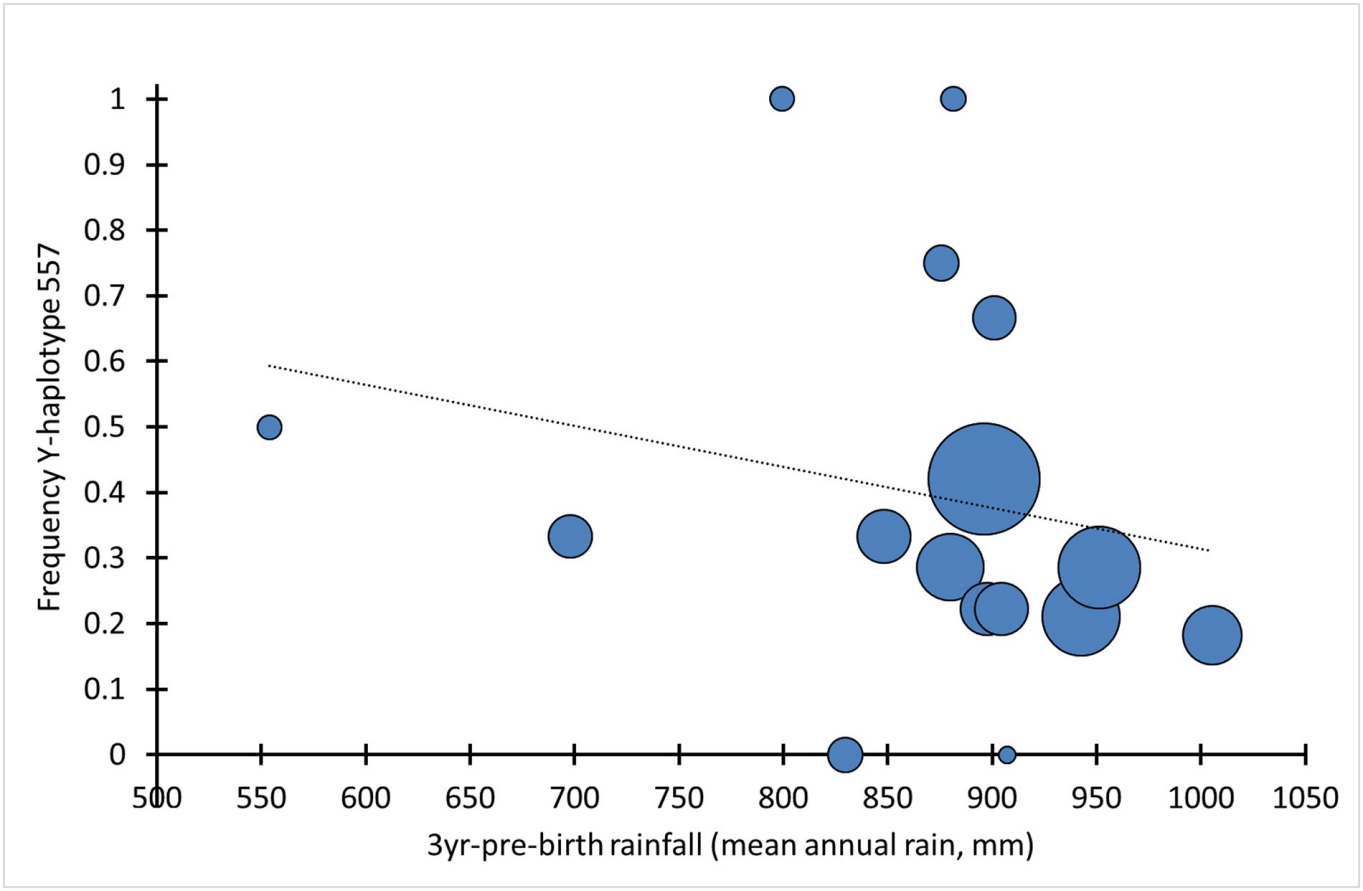
Negative correlation between Y-haplotype-557 frequency and 3-yr-pre-birth rainfall. Area of data points is proportional to sample size, range: 1–38; *ρ* = -0.51, *P* = 0.043, *N*_birth-year-cohorts_ = 16, *N*_individuals_ = 157. Test 6, Table 2.

Although the logistic regression between Y-haplotype status and 3yr-pre-birth rainfall was not significant (*P* = 0.19), there was a significant Spearman correlation between Y-haplotype-557 frequency and 3yr-pre-birth rainfall (*ρ* = -0.51 (weighted by sample size per birth-year cohort), *P* = 0.043; Fig 5, Test 6 in Table 2). This indicates that Y-haplotype 557 is linked to an active sex-ratio suppressor (S1 Text and Table 1).

### Effect of 3yr-pre-birth rainfall on birth sex ratio (research question 4)

**Fig 6:**
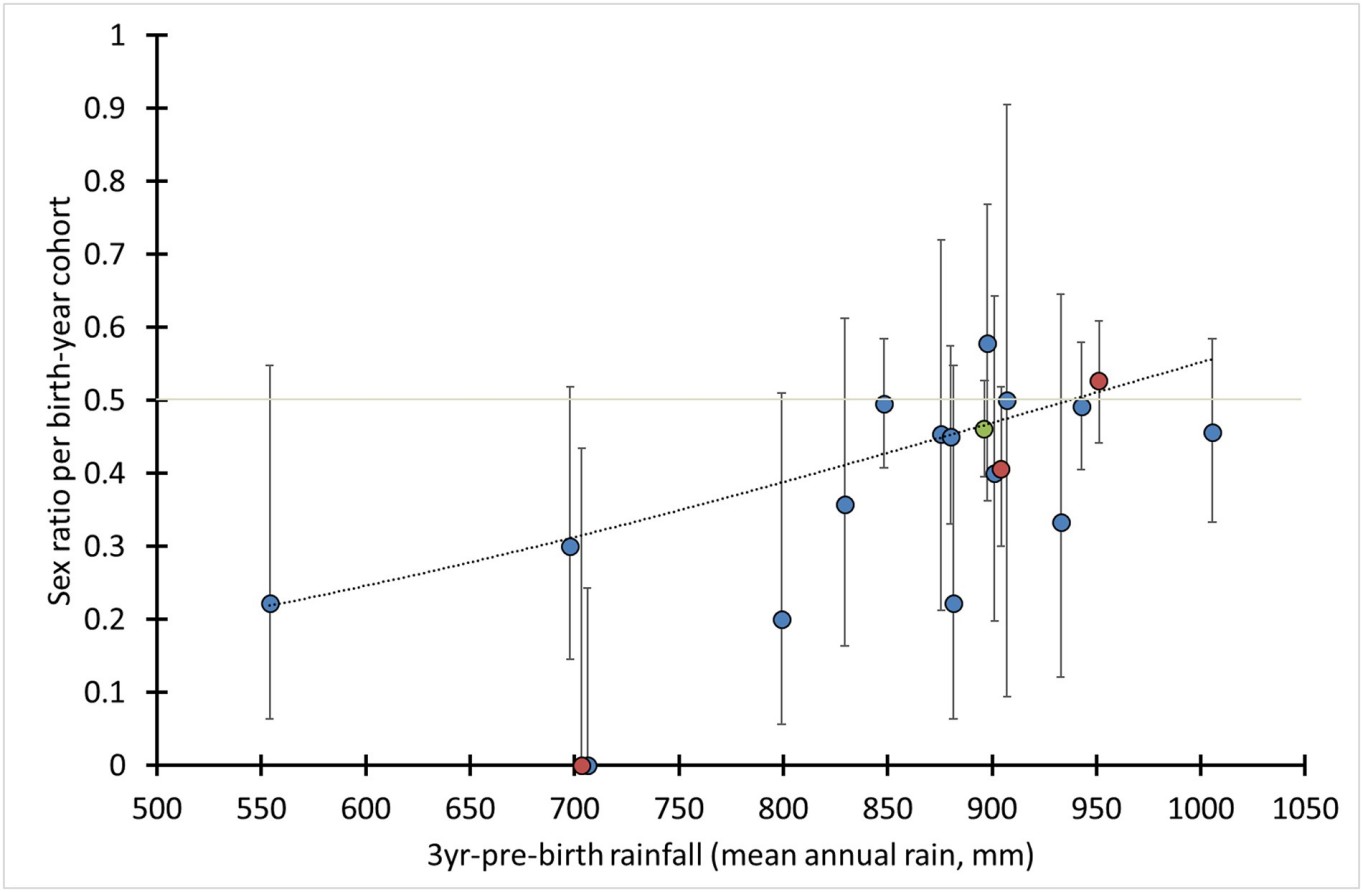
Effect of pre-birth rainfall on sex ratio at birth. Regression line: predicted sex ratio at birth according to logistic regression (*P* = 0.0026), data points: observed sex ratio per birth-year cohort, blue: individuals ≥ 2 years old, red: 0–1 year old calves (at 951 mm: 74 calves and three 2-year olds), green: 126 1-year old calves and 91 2-3-year olds, error bars: 95% CI (Wilson method). Test 7, Table 2.

There was a highly significant positive effect of 3yr-pre-birth rainfall on birth-year cohort specific sex ratios according logistic regression analysis (*P* = 0.0026, *N*_individuals_ = 917; Fig 6, S7 Table; Test 7 in Table 2). The regression equation predicted female-biased birth sex ratios (i.e., at age zero) for 14 out of 17 birth-year cohorts and an average birth sex ratio of 0.43 for the period 1986–2004. Also, when 0-1-year old calves and older individuals were analysed separately, 3yr-pre-birth rainfall had a significant effect (calves: *P* = 0.023, *N*_individuals_ = 335, older individuals: *P* = 0.019, *N*_individuals_ = 582; S7 Table).

## Discussion

The main goal of this study was to find out whether absence of a Y distorter in the HiP buffalo could facilitate BTB-driven negative selection of male-deleterious alleles. We found statistical support to affirmatively answer our four research questions and their underlying hypotheses. We found strong support for negative selection of male-deleterious alleles (Question 1). BTB was probably one of the selective agents, considering that the frequency decrease of microsatellite alleles in HiP relative to northern Kruger was related to their association strength with BTB infection (and low body condition, which in Kruger is associated with BTB). This was further supported by the association between MDL_*s*_ and BTB-infection risk in logistic regression analysis. The interaction between MDL_*s*_ and 3yr-pre-birth rainfall in the latter analysis indicates epigenetic suppression of male-deleterious allele expression after dry 3yr-pre-birth periods (Question 2). The likely occurrence of a Y suppressor at Y-haplotype 557 (Question 3) together with female-biased birth sex ratios (Question 4) support the mechanism depicted in Fig 1. According to this mechanism, BTB causes net positive selection of male-deleterious alleles when a Y distorter is present (as in Kruger) but net negative selection of these alleles when absent (as in HiP).

Four independent findings in relation to 3yr-pre-birth rainfall, a proxy for parental body condition [11], were earlier observed in Kruger: a statistical interaction between 3yr-pre-birth rainfall and MDL_*s*_ (Test 5 in Table 2), and 3yr-pre-birth rainfall correlating with three parameters: microsatellite allele frequencies (Test 4 in Table 2), Y-haplotype-557 frequencies (Test 6 in Table 2) and sex ratio at birth (Test 7 in Table 2). Additionally, we were able to identify two new allele types in HiP with no discernible effect on females, using four independent tests (Tests 2–5 in Table 2): male-specific deleterious and beneficial alleles. The latter alleles were under positive selection and suppressed after wet instead of dry 3yr-pre-birth periods.

### Negative selection of male-deleterious alleles and positive selection of male-beneficial alleles

We observed that the frequency decrease of the SAE_indvO_ alleles in HiP relative to northern Kruger was significantly associated with *A*_sex-anta_, indicative of negative selection against sexually antagonistic alleles or haplotypes of closely linked male-deleterious and female-beneficial alleles. Similarly, the frequency decrease of the DE_indv_ alleles was correlated with *A*_sex-indep_, although non-significantly, providing only weak support for negative selection against alleles deleterious to both sexes. The associations of the SAE_indv_ alleles with 3yr-pre-birth rainfall indicate a selective effect of parental body condition, as was earlier also observed in Kruger [8,9].

Surprisingly, we found a highly significant negative association between *A*_male-spec_ and frequency decrease in HiP among the SAE_indvN_ alleles, which were included in the data analysis as a negative control. Further, birth-year cohort frequencies of SAE_indvN_ alleles were negatively correlated with 3yr-pre-birth rainfall when *A*_male-spec_ > 1, but positively correlated when *A*_male-spec_ < 1. The opposing correlations with 3yr-pre-birth rainfall indicate linkage to two types of male-specific alleles with little or no effect on females: deleterious and under negative selection (low frequencies in HiP) when *A*_male-spec_ > 1, and beneficial and under positive selection (high frequencies in HiP) when *A*_male-spec_ < 1. The male-beneficial effects were supported by the logistic regression analysis (Test 5 in Table 2). The identification of these new allele types suggests that the SAE microsatellites are possibly linked to haplotypes of male-specific and female-specific genes. One haplotype would consist of a male-deleterious and a female-beneficial allele and the other haplotype of a female-deleterious and male-beneficial allele. The male-specificity of the SAE_indvN_ alleles would be due to strong LD with the male-specific alleles, but no or weak LD with the female-specific alleles.

### BTB as selective agent

BTB was likely one of the selective agents, considering that microsatellite-allele frequency decrease in HiP was related to association strength with BTB infection and low body condition in Kruger (the latter being strongly associated with BTB infection) [9], and considering the association between MDL_*s*_ and BTB-infection risk in logistic regression analysis. However, the failure to observe most sexually-antagonistic-linked SAE_indvO,*A*>1_ alleles from Kruger in HiP (Fig 2, S3 Table and S6 Fig) suggests that negative selection has been occurring for many generations already. This could mean that BTB was already present in HiP for a considerable number of years before its discovery in 1986 or that other diseases, such as trypanosomiasis in the 1930s and 1940s, and other environmental factors, such as droughts, provided negative selection pressure as well [22,23].

### Epigenetic effects

The interaction between 3yr-pre-birth rainfall and MDL_*s*_ in logistic regression analysis indicates epigenetic suppression of male-deleterious alleles among animals born after three dry pre-birth years, as was earlier also observed in Kruger [9]. A possible alternative explanation is that the focal alleles are beneficial after dry pre-birth years rather than being suppressed. We consider this less likely as no significant beneficial effects were observed among animals born after dry pre-birth years (*P* = 0.46, S6 Table). Epigenetic suppression of deleterious alleles may be a widespread phenomenon in nature and may diminish the effects of inbreeding depression [29–32].

Surprisingly, there was also an interaction between 3yr-pre-birth rainfall and SAE_indvN,*A*<1_ allele frequencies, indicative of epigenetic suppression of male-specific beneficial alleles, not after three dry but after three wet pre-birth years. This indicates that males born after extended wet multiyear periods have reduced body condition not only because of active deleterious alleles but also because of suppressed beneficial alleles (i.e., reduced relative to males from the same cohort with fewer male-deleterious and male-beneficial alleles). The alternating temporal pattern of epigenetic suppression between male-deleterious and male-beneficial alleles may possibly have evolved to prevent fixation of either the Y distorter or the Y suppressor by reinforcing the epigenetic-driven negative feedback. Such a feedback was earlier hypothesized to be responsible for negative frequency-dependent selection of the Y distorter-suppressor pair [9].

### Possible management implications

The population viability of the HiP buffalo may have been reduced by the high genetic load (alleles at multiple genes with negative fitness effects), despite negative selection of male-deleterious alleles and positive selection of male-beneficial alleles [33]. However, culling is expected to increase the selection pressures on both these allele types. Culling may be made more effective if based on a genotype-and-cull program that targets high-MDL_*s*_ individuals with low frequencies of male-beneficial alleles. Further, targeting males carrying one of the hypothesized sex-ratio distorters would allow removal of the underlying selective agents from the population.

Further improvement of the culling program may be possible by additionally targeting males with active male-deleterious alleles and suppressed male-beneficial alleles, thereby eliminating weak males from the population. Identifying such males may be done by screening of DNA methylation variation [13]. Estimation of MDL_*s*_ and male-beneficial allele frequencies, genotyping of sex chromosomes and screening of DNA methylation variation may also be applied in the commercial breeding of African buffalo (which has become a lucrative business in South Africa), especially with respect to producing disease-free buffalo [34].

Further studies of the gene drive system in the African buffalo may provide new insights for synthetic gene drive systems and their risks. These are being developed to control vector populations of human diseases [1,35]. This study and the ones earlier conducted in Kruger indicate that gene drives can “drive” deleterious alleles into a population not only in close linkage to the driver gene but also genome-wide and that they can influence epigenetic gene regulation.

## Supporting information

S1 Text Sex chromosomal meiotic drive can explain genome-wide high-frequency occurrence of male-deleterious alleles. (DOCX)

S2 Text Frequency differences of DE_majority_ and SAE_pooled_ alleles between HiP and Kruger. (DOCX)

S1 Fig Map with sampling localities. (DOCX)

S2 Fig Monthly rainfall in HiP. (DOCX)

S3 Fig Annual rainfall in HiP in the period 1979–2004. (DOCX)

S4 Fig Frequencies of DE_majority_ and SAE_pooled_ alleles in HiP compared with Kruger. (DOCX)

S5 Fig Difference in *A*_male-spec_ between SAE_indvN_ alleles from Kruger observed and not observed in HIP. (DOCX)

S6 Fig Allele frequency differences between northern Kruger and HiP per SAE allele type. (DOCX)

S1 Table List of SAE_pooled_ and DE_majority_ alleles. (DOCX)

S2 Table List of individual alleles at the DE microsatellite loci. (DOCX)

S3 Table List of individual alleles at the SAE microsatellite loci. (DOCX)

S4 Table List of individual alleles at the SAE microsatellite loci with unknown linkage. (DOCX)

S5 Table Logistic regression of BTB-infection risk for each sex separately. (DOCX)

S6 Table Logistic regression of BTB-infection risk for dry and wet pre-birth years separately. (DOCX)

S7 Table Logistic regression between sex (dependent) and age and 3yr-pre-birth rainfall. (DOCX)

## Abbreviations

*A*_subscript_: indicator of association strength with BTB and body condition in southern Kruger
BTB: bovine tuberculosis
DE: deleterious effect
DE_indv_ alleles: individual alleles at DE microsatellite loci
DE_majority_ alleles: deleterious-effect-associated alleles at DE microsatellite loci, definition according to Van Hooft et al. 2018
HiP: Hluhluwe-iMfolozi Park
MDL: male-deleterious load, definition according to Van Hooft et al. 2018
SAE: sexually-antagonistic effect
SAE_indv_ alleles: individual alleles at SAE microsatellite loci
SAE_indvN_ alleles: individual alleles with no opposite-sex difference at SAE microsatellite loci
SAE_indvO_ alleles: individual alleles with opposite-sex difference at SAE microsatellite loci
SAE_pooled_ alleles: sexually-antagonistic-effect associated alleles at SAE microsatellite loci, definition according to Van Hooft et al. 2018
