# Supplementary material for: A natural gene drive system influences bovine tuberculosis susceptibility in African buffalo: Possible implications for disease management
Source: PLoS One. 2019 Sep 4;14(9):e0221168. doi: 10.1371/journal.pone.0221168 (PMC6726202; doi:10.1371/journal.pone.0221168)
Supplement: S1 Text — (DOCX) [file pone.0221168.s001.docx]

**S1 Text: Sex chromosomal meiotic drive can explain genome-wide high-frequency occurrence of male-deleterious alleles in Kruger buffalo**

Y-haplotype 557 occurred at relatively high frequencies in birth-year cohorts born after dry 3yr-pre-birth periods and in dry-season cohorts (Figures 1 and 4 in [1]). Further, Y-haplotype 557 was directly associated with males carrying relatively many low-body-condition associated microsatellite alleles (Figure 6 in [2]). These two observations combined indicate that Y-haplotype 557 is associated with a Y-chromosomal gene that is mainly active when paternal body condition is low. Low body condition can be induced by low food resource availability (e.g. during droughts) and by male-deleterious alleles. Y-haplotype 112 showed opposite dynamics. It had relatively high frequencies in birth-year cohorts born after wet 3yr-pre-birth periods and in wet-season cohorts (Figures 1 and 4 in [1]). The opposite dynamics indicate that Y-haplotype 112 is associated with a Y-chromosomal gene that is mainly active when paternal body condition is high.

Both Y-chromosomal haplotype frequencies and foetus sex ratios were correlated with pre-birth and seasonal rainfall. Foetus sex ratios were female-biased in foetuses conceived after dry 3yr periods or during dry seasons but male-biased in foetuses conceived after wet 3yr periods or during wet seasons (Figures 2 and 3 in [1]).

One possible explanation for the similarity in rainfall associations between Y-haplotypes and sex-ratio deviations is to assume that the Y-haplotypes are linked to sex-ratio distorters and suppressors [1]. According to this explanation, Y-haplotype 557 is linked to a sex-ratio suppressor because it increases in frequency during rainfall conditions that are associated with female-biased sex ratios (dry pre-birth years and dry seasons). Following the same reasoning, Y-haplotype 112 is linked to a sex-ratio distorter because it increases in frequency during rainfall conditions that are associated with male-biased sex ratios (wet pre-birth years and wet seasons). We think the above explanations are the most likely, given our current knowledge, because meiotic transmission of sex chromosomes has, by necessity, a direct influence on sex ratio. They also explain the high-frequency occurrence of microsatellite alleles associated with deleterious traits only in males (i.e., associated with sexually antagonistic alleles) [2,3], because sex-ratio distorters are only effective in the male sex. Further, they can explain the frequency differences in male-deleterious-trait associated microsatellite alleles between male and female calves (Table 2 in [3]).

In the same population we observed microsatellite alleles that were associated with negative effects on male body condition and health [2,3]. The high frequencies of the microsatellite alleles associated with male-deleterious traits suggest that these alleles were under positive selection. This is supported by the observation of allele-frequency clines with highest allele frequencies where the male-deleterious effects were strongest (in southern Kruger) (Figure 5 in [2]). Positive selection, driven by a sex chromosomal meiotic drive, can explain the counter-intuitive observation that the male-deleterious-trait associated microsatellite alleles had the highest frequencies in birth-year cohorts born after dry 3yr-pre-birth periods (Figure S5 in [2], [3]). This indicates that males with many male-deleterious alleles had the highest reproductive success during droughts, when food resources are relatively scarce. Normally, one would expect strong negative selection of male-deleterious alleles during such periods. The observation that there was also a cline of Y-haplotype 557 provides additional support for a direct involvement of Y-chromosomal genes (Figure S4 in [2]). The Y-cline showed highest frequencies in southern Kruger, just like the male-deleterious-trait associated microsatellite alleles. Thus, the co-occurrence of Y-chromosomal haplotypes and autosomal microsatellite alleles that are both associated with body condition is unlikely to be co-incidental.

We developed a qualitative model that shows that body-condition-associated sex-ratio distorters and suppressors can cause positive selection of male-deleterious alleles. The discovery that male-deleterious allele activity is probably epigenetically regulated in relation to body condition provides strong additional support for our model [3]. We think so, because this epigenetic regulation may cause negative frequency-dependent selection at the Y chromosome, as explained in [3]. Negative frequency-dependent selection seems necessary for stable body-condition-associated Y-chromosomal polymorphism [1]. Otherwise the haplotype with the largest geometric-mean fitness would inevitably sweep to fixation [4]. According to one simulation study, stable non-neutral Y polymorphism only seems possible when assuming sex-ratio distorters and even then only a very small portion of the parameter space admits stability [5]. Interestingly, according to the same simulation study, stable Y polymorphism is only possible when viability and meiotic drive are jointly affected by both X- and Y-linked distorters [5]. We think this is the case with the Kruger buffalo: the occurrence of both an X-distorter (indirectly deduced from the presence of a Y-suppressor) and a Y-distorter, with the activity of both distorters associated with viability via body condition [1,3].

We think alternative explanations that do not involve sex-ratio distorters are less likely and require even more assumptions than our qualitative model. Alternative explanations have to explain the extreme frequency fluctuations in Y-haplotypes between birth-year cohorts (by a factor 5) and between seasons (frequencies of 0.16-0.39 during one season, but none observed in the other season) [1]. Without sex-ratio distorters, these fluctuations are a result of either variation in male reproductive success (related to mating success or semen quality) or sex-specific foetus loss [1]. Variation in male reproductive success as such cannot explain foetus sex-ratio variation or why this sex-ratio variation co-varies with Y-haplotype frequencies on both seasonal and multi-year time scales [1]. Furthermore, mating success and semen quality (in the absence of primary sex-ratio distortion) are polygenic traits which are unlikely dominated by alleles at a single Y-chromosomal gene or haplotype [6,7]. The extreme frequency fluctuations would imply such a dominant effect.

Foetus mortality rates would have to be high to explain the extreme frequency fluctuations in Y-haplotypes. Alternative explanations also have to assume that genetic differences between male and female calves are a result of foetus loss or calf mortality [3]. However, it is difficult to explain the following observations assuming only sex-specific mortality:

- Extreme year-to-year and season-to-season frequency fluctuations in Y-haplotype frequencies that do not result in extinction or fixation of one of the Y-haplotypes due to genetic drift [1].
- High pregnancy rate (70%) [1]. This is difficult to reconcile with high foetus mortality. Please note that sex-ratio distortion genes are expected to only affect male fertility [8,9].
- Absence of any appreciable difference in the fraction of high-body-condition animals between male and female calves [3]. This is difficult to reconcile with sex-specific calf mortality.
- Individuals with many male-deleterious alleles having the highest reproductive success during droughts (i.e., high frequencies of male-deleterious-trait associated microsatellite alleles in birth-year cohorts born after dry 3yr-pre-birth rainfall periods) [2,3].
- Absence of significant ‘microsatellite allele-deleterious trait’ associations in animals born during droughts (i.e. ‘genetic-measure by 3yr-pre-birth-rainfall’ interactions in logistic regression analyses with deleterious trait as dependent variable) [3].
- The co-occurrence of two types of allele-frequency clines: one based on Y-haplotype 557 and the other based on male-deleterious-trait associated microsatellite alleles [1,2].
- Direct association between Y-haplotype 557 and homozygous microsatellites alleles associated with low body condition [2].

Various other alternative explanations have been discussed in earlier publications ([1,3], Text S1 in [2]).

**References**

1. van Hooft P, Prins HHT, Getz WM, Jolles AE, van Wieren SE, Greyling BJ, et al. (2010) Rainfall-driven sex-ratio genes in African buffalo suggested by correlations between Y-chromosomal haplotype frequencies and foetal sex ratio. BMC Evol Biol 10: 10.1186/1471-2148-1110-1106.

2. van Hooft P, Greyling BJ, Getz WM, van Helden PD, Zwaan BJ, Bastos ADS (2014) Positive selection of deleterious alleles through interaction with a sex-ratio suppressor gene in African buffalo: a plausible new mechanism for a high frequency anomaly. PLoS ONE 9: e111778.

3. Van Hooft P, Dougherty ER, Getz WM, Greyling BJ, Zwaan BJ, Bastos ADS (2018) Genetic responsiveness of African buffalo to environmental stressors: a role for epigenetics in balancing autosomal and sex chromosome interactions? PLoS ONE.

4. Dean AM (2005) Protecting haploid polymorphisms in temporally variable environments. Genetics 169: 1147-1156.

5. Clark AG (1987) Natural selection and Y-linked polymorphism. Genetics 115: 569-577.

6. Sarakul M, Elzo MA, Koonawootrittriron S, Suwanasopee T, Jattawa D (2018) Genetic parameters, predictions, and rankings for semen production traits in a Thailand multi-breed dairy population using genomic-polygenic and polygenic models. Anim Reprod Sci 195: 71-79.

7. Hughes KA, Leips J (2006) Quantitative trait locus analysis of male mating success and sperm competition in Drosophila melanogaster. Evolution 60: 1427-1434.

8. Jaenike J (2001) Sex chromosome meiotic drive. Annu Rev Ecol Syst 32: 25-49.

9. Price TAR, Wedell N (2008) Selfish genetic elements and sexual selection: their impact on male fertility. Genetica 132: 295-307.
