# Supplementary material for: A natural gene drive system influences bovine tuberculosis susceptibility in African buffalo: Possible implications for disease management
Source: PLoS One. 2019 Sep 4;14(9):e0221168. doi: 10.1371/journal.pone.0221168 (PMC6726202; doi:10.1371/journal.pone.0221168)
Supplement: S2 Text — (DOCX) [file pone.0221168.s002.docx]

**S2 Text. Frequency differences of DE_majority_ and SAE_pooled_ alleles between HiP and Kruger.**

The average frequency of DE_majority_ and SAE_pooled_ alleles in HiP non-significantly decreased by 19% relative to Kruger (average frequency HiP = 0.54, Kruger = 0.66, Wilcoxon signed-rank test: *P* = 0.19; Figure S4). Five alleles even increased in frequency. Half of the frequency decrease was due to two rare alleles in HiP with a frequency < 0.03 (on average 10% decrease when excluding these alleles). Allele frequency variation was significantly larger in HiP than in Kruger, even when excluding the two rare alleles, which indicates relatively strong genetic drift in HiP (Levene’s test: *F* = 14.2, *P* = 0.00067; excluding the two rare alleles: *F* = 10.9, *P* = 0.0026).

Non-significance of the frequency decrease may have been due to large between-locus allele-frequency variation in HiP, indicating strong genetic drift as a result of the population bottleneck between 1895 and 1929, and low statistical power because of low allele number (only one per locus) and relatively weak linkage between SAE_pooled_ alleles and sexually antagonistic alleles. The latter is supported by the observation of a frequency increase of two SAE_pooled_ alleles, but a frequency decrease of all seven SAE_indvO_ alleles at the same loci (*ILSTS026* and *TLGA159*).
