# Supplementary material for: A natural gene drive system influences bovine tuberculosis susceptibility in African buffalo: Possible implications for disease management
Source: PLoS One. 2019 Sep 4;14(9):e0221168. doi: 10.1371/journal.pone.0221168 (PMC6726202; doi:10.1371/journal.pone.0221168)
Supplement: S1 Fig — (DOCX) [file pone.0221168.s003.docx]

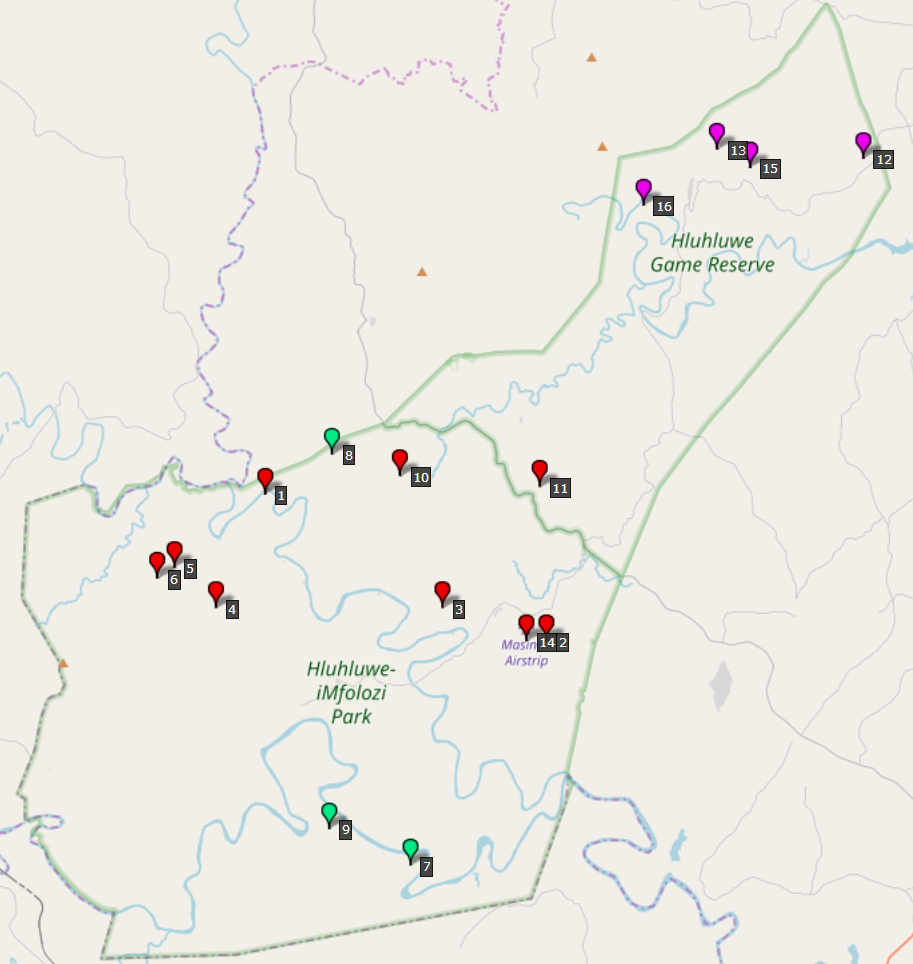


S1 Fig. Map with sampling localities.

Herds 1-13: genotyped herds, red: sampled in 2002, green: sampled in 2003, magenta: sampled in 2004. © OpenStreetMap contributors. The data is available under the Open Database License, and the cartography is licensed as CC BY-SA.
