# Supplementary figures and images for: A natural gene drive system influences bovine tuberculosis susceptibility in African buffalo: Possible implications for disease management
Source: PLoS One. 2019 Sep 4;14(9):e0221168. doi: 10.1371/journal.pone.0221168 (PMC6726202; doi:10.1371/journal.pone.0221168)

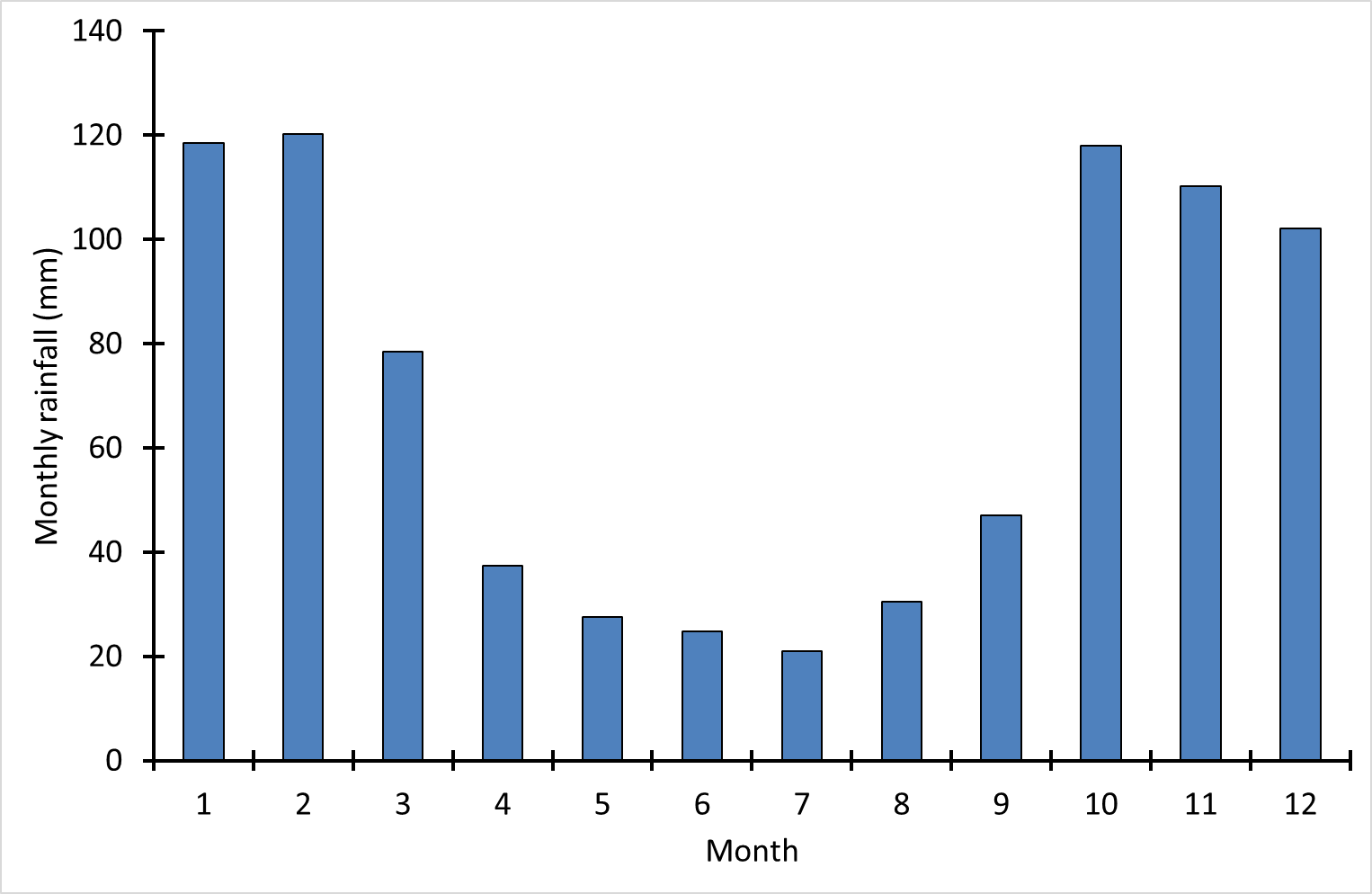


S2 Fig. Monthly rainfall in HiP.

Supplement: S2 Fig — (DOCX) [file pone.0221168.s004.docx]
