# Supplementary material for: A natural gene drive system influences bovine tuberculosis susceptibility in African buffalo: Possible implications for disease management
Source: PLoS One. 2019 Sep 4;14(9):e0221168. doi: 10.1371/journal.pone.0221168 (PMC6726202; doi:10.1371/journal.pone.0221168)
Supplement: S3 Fig — (DOCX) [file pone.0221168.s005.docx]

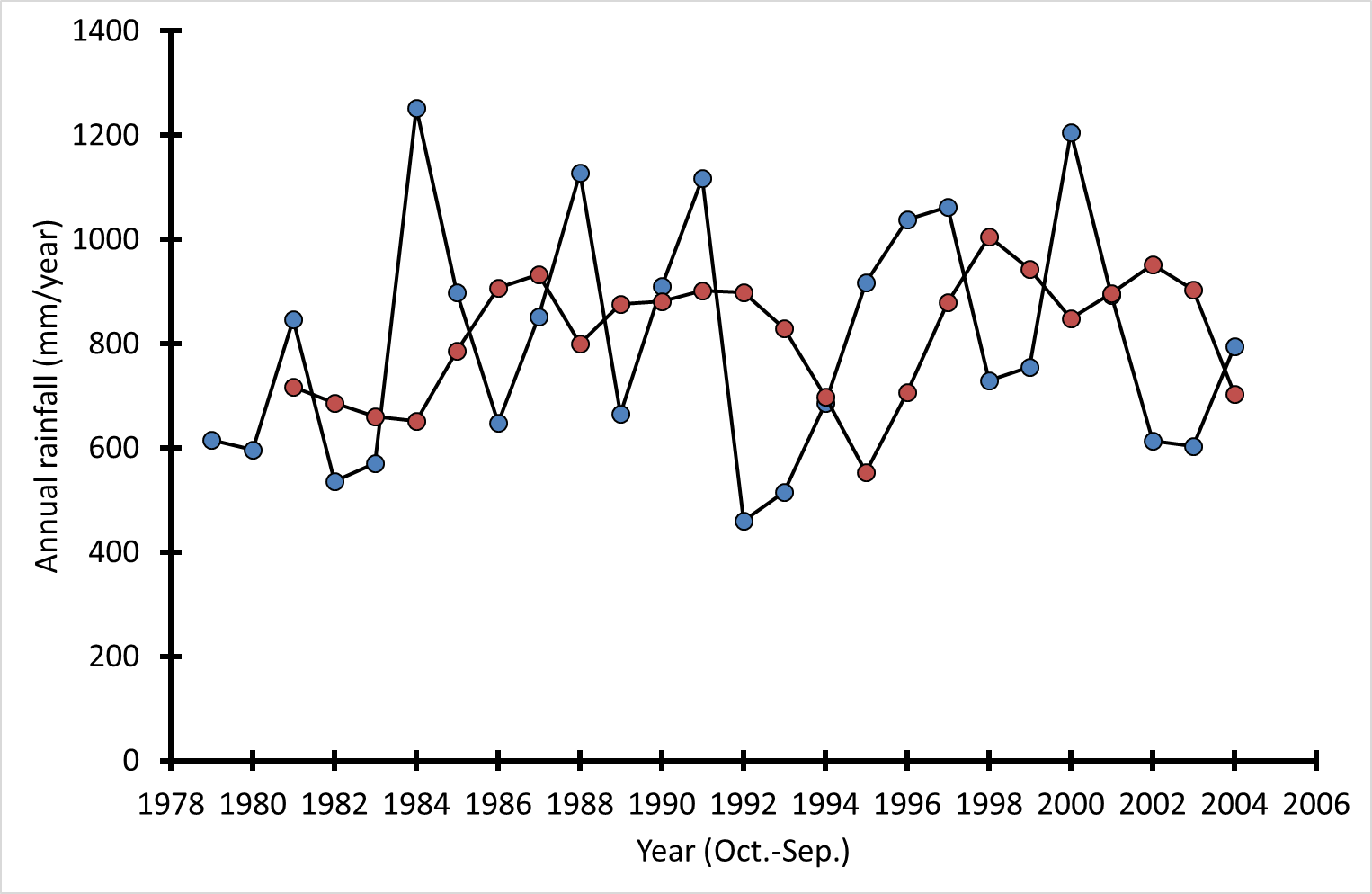


S3 Fig. Annual rainfall in HiP in the period 1979-2004.

Blue data points: annual rainfall (years running from October to September), red data points: average annual rainfall in the three years before the year of birth
