# Supplementary material for: A natural gene drive system influences bovine tuberculosis susceptibility in African buffalo: Possible implications for disease management
Source: PLoS One. 2019 Sep 4;14(9):e0221168. doi: 10.1371/journal.pone.0221168 (PMC6726202; doi:10.1371/journal.pone.0221168)
Supplement: S4 Fig — (DOCX) [file pone.0221168.s006.docx]

*
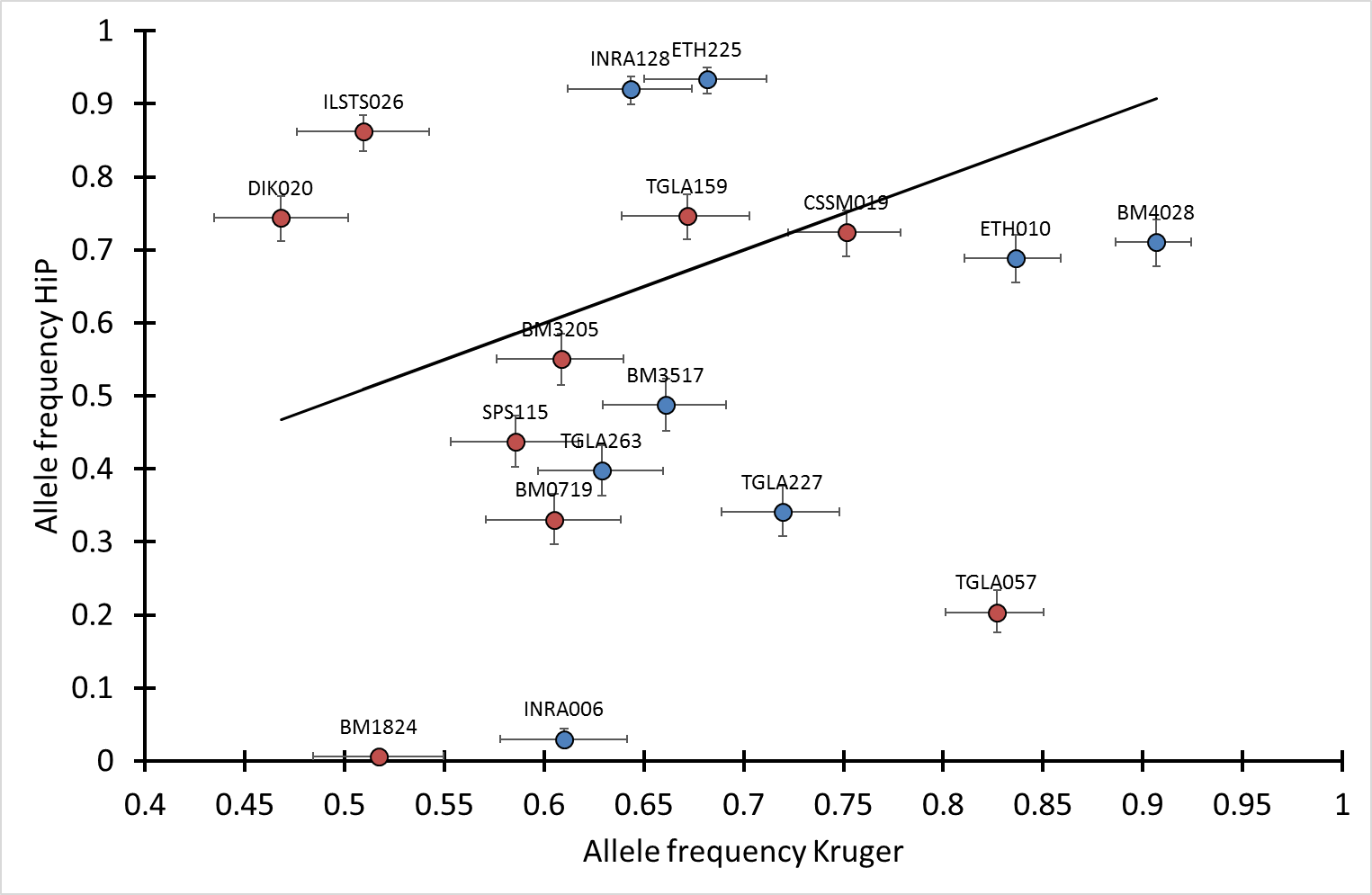
*

S4 Fig. Frequencies of DE_majority_ and SAE_pooled_ alleles in HiP compared with Kruger.

Red data points: SAE_pooled_ alleles, blue data points: DE_majority_ alleles, error bars: 95% CI (Wilson method), data labels: microsatellite name, line: frequency HiP = frequency Kruger, *N*_individuals_ HiP = 373-401, *N*_individuals_ Kruger = 395-458.
