# Supplementary material for: A natural gene drive system influences bovine tuberculosis susceptibility in African buffalo: Possible implications for disease management
Source: PLoS One. 2019 Sep 4;14(9):e0221168. doi: 10.1371/journal.pone.0221168 (PMC6726202; doi:10.1371/journal.pone.0221168)
Supplement: S5 Fig — (DOCX) [file pone.0221168.s007.docx]

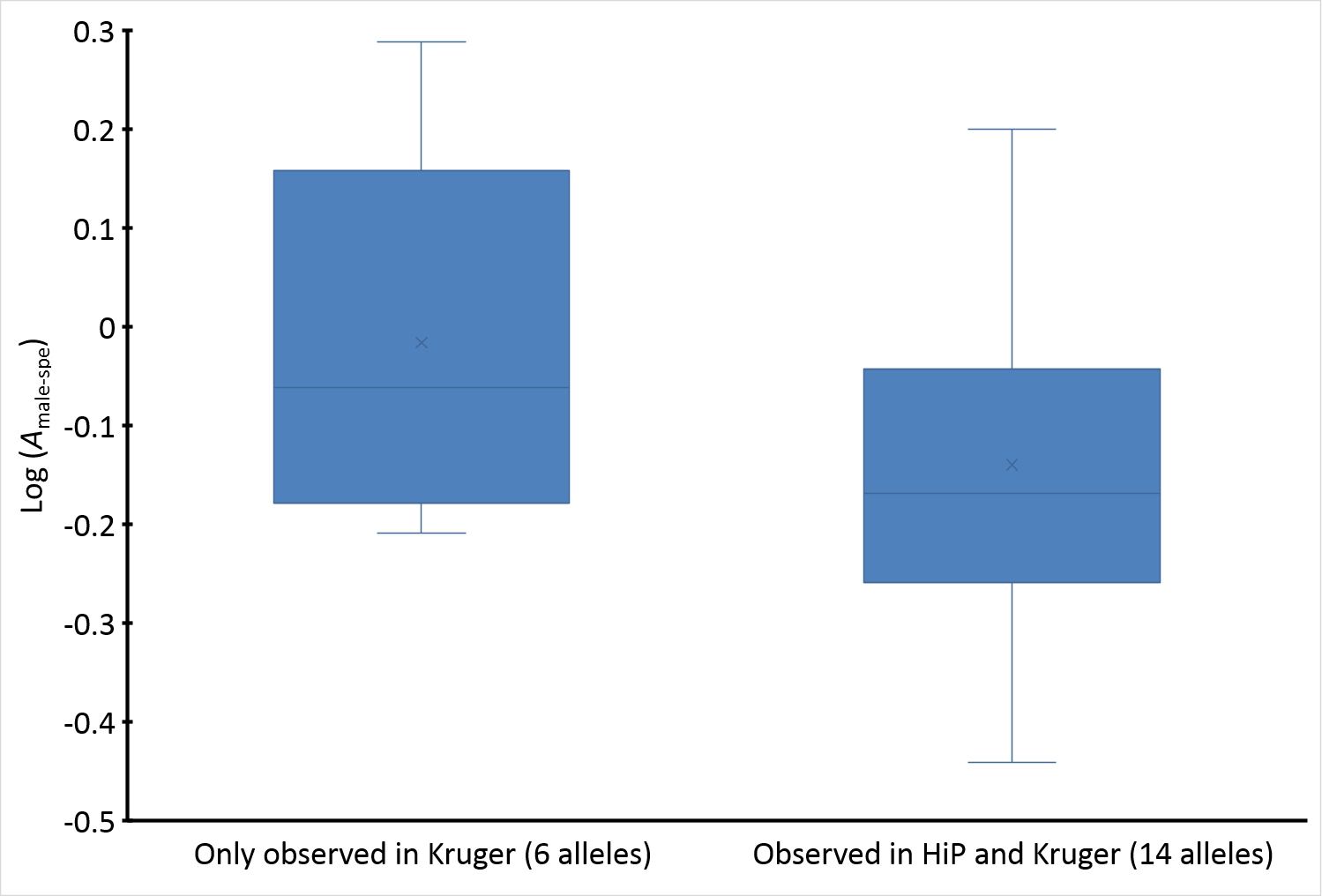
 S5 Fig. Difference in *A*_male-spec_ between SAE_indvN_ alleles from Kruger observed and not observed in HiP

Median difference = 0.08, *P* = 0.15.
