# Supplementary material for: A natural gene drive system influences bovine tuberculosis susceptibility in African buffalo: Possible implications for disease management
Source: PLoS One. 2019 Sep 4;14(9):e0221168. doi: 10.1371/journal.pone.0221168 (PMC6726202; doi:10.1371/journal.pone.0221168)
Supplement: S6 Fig — (DOCX) [file pone.0221168.s008.docx]

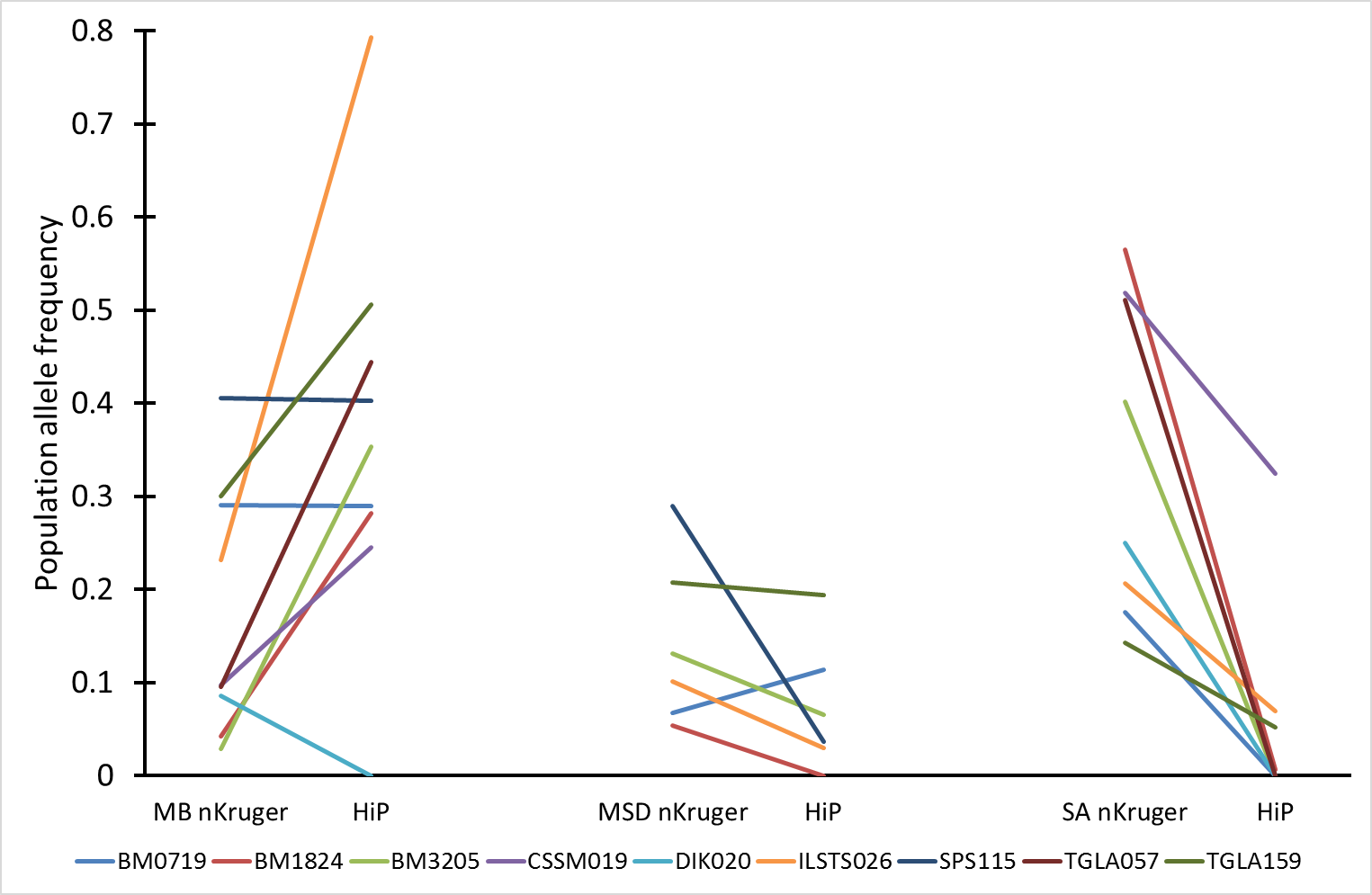


S6 Fig. Allele frequency differences between northern Kruger and HiP per SAE allele type.

Y-axis: total frequency per locus (i.e. pooled all alleles of a certain allele type), MB: male-beneficial linked microsatellite alleles (SAE_indvN-_*_A_*_<1_ alleles), MSD: male-specific-deleterious linked microsatellite alleles (SAE_indvN-_*_A_*_>1_), SA: sexually-antagonistic linked microsatellite alleles (SAE_indvO-_*_A_*_>1_).
