# Supplementary material for: A natural gene drive system influences bovine tuberculosis susceptibility in African buffalo: Possible implications for disease management
Source: PLoS One. 2019 Sep 4;14(9):e0221168. doi: 10.1371/journal.pone.0221168 (PMC6726202; doi:10.1371/journal.pone.0221168)
Supplement: S1 Table — (DOCX) [file pone.0221168.s009.docx]

S1 Table. List of SAE_pooled_ and DE_majority_ alleles.

*: also included as SAE_indvO-_*_A_*_>1_ allele (sexually antagonistic, 12 out of 27), ^: also included as SAE_indvN-_*_A_*_>1_ allele (male-specific deleterious, 3 out of 27), $: also included as SAE_indvN-_*_A_*_<1_ allele (male-beneficial, 7 out of 27), #: also included as DE_indv_ allele (DE, 7 out of 8).

| Locus | Allele size | Freq. HiP | Freq. Kruger | Allele type |
| --- | --- | --- | --- | --- |
| BM0719 | 148 | 0.040 | 0.300 | SAE_pooled_ |
| BM0719 | 152* | Not observed | 0.142 | SAE_pooled_ |
| BM0719 | 154$ | 0.290 | 0.163 | SAE_pooled_ |
| BM1824 | 181* | 0.006 | 0.295 | SAE_pooled_ |
| BM1824 | 187* | Not observed | 0.100 | SAE_pooled_ |
| BM1824 | 197* | Not observed | 0.122 | SAE_pooled_ |
| BM3205 | 202 | 0.485 | 0.205 | SAE_pooled_ |
| BM3205 | 206* | Not observed | 0.215 | SAE_pooled_ |
| BM3205 | 214^ | 0.065 | 0.189 | SAE_pooled_ |
| CSSM19 | 144* | 0.324 | 0.383 | SAE_pooled_ |
| CSSM19 | 146* | Not observed | 0.173 | SAE_pooled_ |
| CSSM19 | 148 | 0.405 | 0.192 | SAE_pooled_ |
| DIK20 | 164$ | Not observed | 0.136 | SAE_pooled_ |
| DIK20 | 184 | 0.746 | 0.150 | SAE_pooled_ |
| DIK20 | 198* | Not observed | 0.182 | SAE_pooled_ |
| IILSTS26 | 149* | 0.069 | 0.197 | SAE_pooled_ |
| IILSTS26 | 151$ | Not observed | 0.153 | SAE_pooled_ |
| IILSTS26 | 163$ | 0.793 | 0.159 | SAE_pooled_ |
| SPS115 | 235$ | 0.351 | 0.149 | SAE_pooled_ |
| SPS115 | 237^ | 0.037 | 0.120 | SAE_pooled_ |
| SPS115 | 239$ | 0.052 | 0.317 | SAE_pooled_ |
| TGLA057 | 93* | Not observed | 0.275 | SAE_pooled_ |
| TGLA057 | 95* | Not observed | 0.257 | SAE_pooled_ |
| TGLA057 | 97 | 0.200 | 0.295 | SAE_pooled_ |
| TGLA159 | 223^ | 0.194 | 0.208 | SAE_pooled_ |
| TGLA159 | 227$ | 0.506 | 0.319 | SAE_pooled_ |
| TGLA159 | 231* | 0.052 | 0.145 | SAE_pooled_ |
| BM3517 | 92# | 0.490 | 0.661 | DE_majority_ |
| BM4028 | 134# | 0.712 | 0.907 | DE_majority_ |
| ETH010 | 204# | 0.688 | 0.836 | DE_majority_ |
| ETH225 | 133# | 0.935 | 0.682 | DE_majority_ |
| INRA006 | 113 | 0.029 | 0.610 | DE_majority_ |
| INRA128 | 176# | 0.924 | 0.643 | DE_majority_ |
| TGLA227 | 72# | 0.341 | 0.719 | DE_majority_ |
| TGLA263 | 122# | 0.398 | 0.629 | DE_majority_ |
