# Supplementary material for: A natural gene drive system influences bovine tuberculosis susceptibility in African buffalo: Possible implications for disease management
Source: PLoS One. 2019 Sep 4;14(9):e0221168. doi: 10.1371/journal.pone.0221168 (PMC6726202; doi:10.1371/journal.pone.0221168)
Supplement: S2 Table — (DOCX) [file pone.0221168.s010.docx]

S2 Table. List of individual alleles at the DE microsatellite loci.

DE: deleterious allele, ?: possibly linked to a wild-type allele or to a less deleterious alternative allele, but this could not be tested. All alleles observed ≥ 15 times in southern Kruger.

| Locus | Allele size (bp) | Freq. HiP | Freq. northern Kruger | Freq.  southern Kruger | Allele type | Linked to | *A*_sex-indep_ |
| --- | --- | --- | --- | --- | --- | --- | --- |
| BM3517 | 92 | 0.490 | 0.639 | 0.671 | DE_indv_ | DE | 1.04 |
| BM4028 | 134 | 0.712 | 0.886 | 0.916 | DE_indv_ | DE | 1.07 |
| ETH010 | 204 | 0.688 | 0.800 | 0.852 | DE_indv_ | DE | 1.17 |
| ETH225 | 133 | 0.935 | 0.689 | 0.678 | DE_indv_ | DE | 1.14 |
| INRA128 | 176 | 0.924 | 0.622 | 0.653 | DE_indv_ | DE | 1.16 |
| TGLA227 | 72 | 0.341 | 0.727 | 0.716 | DE_indv_ | DE | 1.08 |
| TGLA263 | 122 | 0.398 | 0.609 | 0.638 | DE_indv_ | DE | 1.09 |
| BM3517 | 86 | 0.052 | 0.073 | 0.102 | DE_indv_ | ? | 0.55 |
| BM4028 | 132 | 0.259 | 0.063 | 0.067 | DE_indv_ | ? | 0.32 |
| ETH010 | 206 | 0.312 | 0.193 | 0.147 | DE_indv_ | ? | 0.40 |
| ETH225 | 137 | 0.065 | 0.307 | 0.320 | DE_indv_ | ? | 0.74 |
| INRA006 | 115 | 0.338 | 0.149 | 0.147 | DE_indv_ | ? | 0.91 |
| INRA128 | 166 | Not observed | 0.078 | 0.075 | DE_indv_ | ? | 0.55 |
| INRA128 | 168 | Not observed | 0.100 | 0.058 | DE_indv_ | ? | 0.32 |
| TGLA227 | 76 | 0.316 | 0.068 | 0.085 | DE_indv_ | ? | 0.53 |
| TGLA263 | 124 | 0.390 | 0.250 | 0.198 | DE_indv_ | ? | 0.79 |
