# Supplementary material for: A natural gene drive system influences bovine tuberculosis susceptibility in African buffalo: Possible implications for disease management
Source: PLoS One. 2019 Sep 4;14(9):e0221168. doi: 10.1371/journal.pone.0221168 (PMC6726202; doi:10.1371/journal.pone.0221168)
Supplement: S3 Table — (DOCX) [file pone.0221168.s011.docx]

S3 Table. List of individual alleles at the SAE microsatellite loci.

*: also included in SAE_pooled_ alleles, #: *A*_sex-anta_ in case of SAE_indvO_ alleles, *A*_male-spec_ in case of SAE_indvN_ alleles. All alleles observed ≥ 15 times in southern Kruger. SA: sexually antagonistic allele, MSD: male-specific deleterious allele, MB: male-beneficial allele (male-beneficial).

| Locus | Allele size | Freq. HiP | Freq. northern Kruger | Freq.  southern Kruger | Allele type | Linked to | *A^#^* |
| --- | --- | --- | --- | --- | --- | --- | --- |
| BM0719 | 152 | Not observed | 0.175 | 0.125 | SAE_indvO-_*_A_*_>1_ | SA | 1.76 |
| BM1824 | 181 | 0.006 | 0.373 | 0.262 | SAE_indvO-_*_A_*_>1_ | SA | 1.26 |
| BM1824 | 187 | Not observed | 0.100 | 0.101 | SAE_indvO-_*_A_*_>1_ | SA | 2.58 |
| BM1824 | 197 | Not observed | 0.092 | 0.134 | SAE_indvO-_*_A_*_>1_ | SA | 1.19 |
| BM3205 | 204 | Not observed | 0.153 | 0.149 | SAE_indvO-_*_A_*_>1_ | SA | 1.43 |
| BM3205 | 206 | Not observed | 0.248 | 0.200 | SAE_indvO-_*_A_*_>1_ | SA | 1.35 |
| CSSM19 | 144 | 0.324 | 0.328 | 0.407 | SAE_indvO-_*_A_*_>1_ | SA | 1.32 |
| CSSM19 | 146 | Not observed | 0.190 | 0.166 | SAE_indvO-_*_A_*_>1_ | SA | 1.35 |
| DIK20 | 188 | Not observed | 0.123 | 0.113 | SAE_indvO-_*_A_*_>1_ | SA | 1.38 |
| DIK20 | 198 | Not observed | 0.127 | 0.204 | SAE_indvO-_*_A_*_>1_ | SA | 1.19 |
| IILSTS26 | 149 | 0.069 | 0.207 | 0.193 | SAE_indvO-_*_A_*_>1_ | SA | 1.48 |
| TGLA057 | 93 | Not observed | 0.243 | 0.289 | SAE_indvO-_*_A_*_>1_ | SA | 1.23 |
| TGLA057 | 95 | Not observed | 0.268 | 0.252 | SAE_indvO-_*_A_*_>1_ | SA | 1.37 |
| TGLA159 | 231 | 0.052 | 0.142 | 0.146 | SAE_indvO-_*_A_*_>1_ | SA | 2.17 |
| BM0719 | 136 | 0.114 | 0.067 | 0.107 | SAE_indvN-_*_A_*_>1_ | MSD | 1.29 |
| BM1824 | 185 | Not observed | 0.054 | 0.088 | SAE_indvN-_*_A_*_>1_ | MSD | 1.40 |
| BM3205 | 214 | 0.065 | 0.131 | 0.214 | SAE_indvN-_*_A_*_>1_ | MSD | 1.28 |
| IILSTS26 | 159 | 0.029 | 0.101 | 0.116 | SAE_indvN-_*_A_*_>1_ | MSD | 1.19 |
| SPS115 | 223 | Not observed | 0.047 | 0.063 | SAE_indvN-_*_A_*_>1_ | MSD | 4.47 |
| SPS115 | 229 | Not observed | 0.112 | 0.091 | SAE_indvN-_*_A_*_>1_ | MSD | 2.01 |
| SPS115 | 237 | 0.037 | 0.130 | 0.115 | SAE_indvN-_*_A_*_>1_ | MSD | 1.34 |
| TGLA159 | 223 | 0.194 | 0.208 | 0.209 | SAE_indvN-_*_A_*_>1_ | MSD | 1.46 |
| BM0719 | 146 | Not observed | 0.149 | 0.125 | SAE_indvN-_*_A_*_<1_ | MB | 0.58 |
| BM0719 | 154 | 0.290 | 0.142 | 0.174 | SAE_indvN-_*_A_*_<1_ | MB | 0.96 |
| BM1824 | 183 | 0.282 | 0.042 | 0.086 | SAE_indvN-_*_A_*_<1_ | MB | 0.72 |
| BM3205 | 212 | 0.353 | 0.029 | 0.057 | SAE_indvN-_*_A_*_<1_ | MB | 0.43 |
| CSSM19 | 150 | 0.245 | 0.097 | 0.052 | SAE_indvN-_*_A_*_<1_ | MB | 0.56 |
| DIK20 | 164 | Not observed | 0.086 | 0.156 | SAE_indvN-_*_A_*_<1_ | MB | 0.31 |
| IILSTS26 | 151 | Not observed | 0.054 | 0.200 | SAE_indvN-_*_A_*_<1_ | MB | 0.95 |
| IILSTS26 | 163 | 0.793 | 0.178 | 0.150 | SAE_indvN-_*_A_*_<1_ | MB | 0.97 |
| SPS115 | 235 | 0.351 | 0.112 | 0.165 | SAE_indvN-_*_A_*_<1_ | MB | 0.91 |
| SPS115 | 239 | 0.052 | 0.293 | 0.327 | SAE_indvN-_*_A_*_<1_ | MB | 0.98 |
| TGLA057 | 99 | 0.445 | 0.096 | 0.068 | SAE_indvN-_*_A_*_<1_ | MB | 0.90 |
| TGLA159 | 227 | 0.506 | 0.300 | 0.327 | SAE_indvN-_*_A_*_<1_ | MB | 0.97 |
