# Supplementary material for: A natural gene drive system influences bovine tuberculosis susceptibility in African buffalo: Possible implications for disease management
Source: PLoS One. 2019 Sep 4;14(9):e0221168. doi: 10.1371/journal.pone.0221168 (PMC6726202; doi:10.1371/journal.pone.0221168)
Supplement: S4 Table — (DOCX) [file pone.0221168.s012.docx]

S4 Table. List of individual alleles at the SAE microsatellite loci with unknown linkage.

?: probably not linked to a specific allele type at a coding gene. All alleles observed ≥ 15 times in southern Kruger.

| Locus | Allele size | Freq. HiP | Freq. northern Kruger | Freq.  southern Kruger | Allele type | Linked to | *A*_sex-anta_ |
| --- | --- | --- | --- | --- | --- | --- | --- |
| BM0719 | 148 | 0.040 | 0.261 | 0.320 | SAE_indvO-_*_A_*_<1_ | ? | 0.80 |
| BM1824 | 195 | 0.019 | 0.054 | 0.083 | SAE_indvO-_*_A_*_<1_ | ? | 0.71 |
| BM3205 | 198 | 0.097 | 0.069 | 0.062 | SAE_indvO-_*_A_*_<1_ | ? | 0.53 |
| BM3205 | 202 | 0.485 | 0.208 | 0.203 | SAE_indvO-_*_A_*_<1_ | ? | 0.84 |
| BM3205 | 208 | Not observed | 0.073 | 0.065 | SAE_indvO-_*_A_*_<1_ | ? | 0.49 |
| CSSM19 | 148 | 0.405 | 0.164 | 0.204 | SAE_indvO-_*_A_*_<1_ | ? | 0.58 |
| DIK20 | 184 | 0.746 | 0.209 | 0.126 | SAE_indvO-_*_A_*_<1_ | ? | 0.61 |
| DIK20 | 186 | 0.082 | 0.074 | 0.108 | SAE_indvO-_*_A_*_<1_ | ? | 0.72 |
| IILSTS26 | 153 | 0.013 | 0.141 | 0.138 | SAE_indvO-_*_A_*_<1_ | ? | 0.90 |
| IILSTS26 | 157 | 0.096 | 0.163 | 0.101 | SAE_indvO-_*_A_*_<1_ | ? | 0.74 |
| SPS115 | 227 | 0.155 | 0.025 | 0.041 | SAE_indvO-_*_A_*_<1_ | ? | 0.50 |
| SPS115 | 249 | 0.055 | 0.033 | 0.064 | SAE_indvO-_*_A_*_<1_ | ? | 0.82 |
| TGLA057 | 89 | 0.030 | 0.040 | 0.056 | SAE_indvO-_*_A_*_<1_ | ? | 0.57 |
| TGLA057 | 97 | 0.200 | 0.305 | 0.290 | SAE_indvO-_*_A_*_<1_ | ? | 0.71 |
| TGLA159 | 225 | 0.100 | 0.112 | 0.126 | SAE_indvO-_*_A_*_<1_ | ? | 0.52 |
| TGLA159 | 229 | 0.033 | 0.035 | 0.054 | SAE_indvO-_*_A_*_<1_ | ? | 0.80 |
| TGLA159 | 235 | 0.115 | 0.142 | 0.101 | SAE_indvO-_*_A_*_<1_ | ? | 0.65 |
