# Supplementary material for: A natural gene drive system influences bovine tuberculosis susceptibility in African buffalo: Possible implications for disease management
Source: PLoS One. 2019 Sep 4;14(9):e0221168. doi: 10.1371/journal.pone.0221168 (PMC6726202; doi:10.1371/journal.pone.0221168)
Supplement: S5 Table — (DOCX) [file pone.0221168.s013.docx]

S5 Table. Logistic regression of BTB-infection risk for each sex separately.

Males: *N*_BTB-positive_ = 17, *N*_BTB-negative_ = 141, EPV = 3.4, Pearson correlation between main factors: |*r*| ≤ 0.53; females: *N*_BTB-positive_ = 30, *N*_BTB-negative_ = 194, EPV = 6.0, AIC = 173.6, Pearson correlation between main factors: *r* = -0.028, SAE_i-non-OSD<1_-by-pre-birth rainfall interaction was not significant when included in the model and resulted in a considerable increase of AIC (*P* = 0.84, AIC = 177.4). a: Combined *P* value for both sexes = 0.65, b: combined *P* value for both sexes = 0.035. Combined *P* values were estimated with the Z-transform test.

Logistic regression analysis was implemented the `lme4' package (version 1.1.13) in R. Herd affiliation and sampling year were incorporated as a random intercept in a mixed modelling approach. To aid in regression model convergence, all continuous variables were scaled per sex by subtracting the mean of each variable from each observation and dividing the result by the standard deviation of that variable.

| Parameter | Mean | SE | *P* value | Mean | SE | *P* value |
| --- | --- | --- | --- | --- | --- | --- |
|  | Males | | | Females | | |
| MDL (scaled; *x*_1_) | -0.047 | 0.368 | 0.898^a^ | -0.117 | 0.226 | 0.603^a^ |
| SAE_indvN-_*_A_*_<1_ (scaled; *x*_2_) | -0.842 | 0.398 | 0.034 |  |  |  |
| Pre-birth rainfall (scaled; *x*_3_) | 0.632 | 0.422 | 0.134 | -0.271 | 0.196 | 0.167 |
| *x*_1_-by-*x*_3_ interaction | 0.689 | 0.390 | 0.078^b^ | 0.247 | 0.204 | 0.226^b^ |
| *x*_2_-by-*x*_3_ interaction | 1.526 | 0.548 | 0.0053 |  |  |  |
| Intercept | -3.102 | 0.838 | 0.0002 | -2.547 | 1.183 | 0.031 |
