# Supplementary material for: A natural gene drive system influences bovine tuberculosis susceptibility in African buffalo: Possible implications for disease management
Source: PLoS One. 2019 Sep 4;14(9):e0221168. doi: 10.1371/journal.pone.0221168 (PMC6726202; doi:10.1371/journal.pone.0221168)
Supplement: S6 Table — (DOCX) [file pone.0221168.s014.docx]

S6 Table: Logistic regression of BTB-infection risk for dry and wet pre-birth years separately.

Males wet years/females wet years/males dry years/females dry years: *N*_BTB-positive_ = 9, 12, 8, 18; *N*_BTB-negative_ = 67, 70, 74, 124; EPV = 4.5, 12, 4.0, 18; Pearson correlation between main factors: males wet years: *r* = 0.19, males dry years: *r* = 0.38. Wet and dry years: respectively > 897 and < 897 mm mean annual rainfall in the three years before the year of birth. a: Combined *P* value for both sexes = 0.081, b: combined *P* value for both sexes = 0.464. Combined *P* values were estimated with the Z-transform test.

Logistic regression analysis was implemented the `lme4' package (version 1.1.13) in R. Only herd affiliation was incorporated as a random intercept in a mixed modelling approach, because some models failed to converge when also sampling year was included as random factor. Gauss-Hermite quadrature was used for parameter estimation. The continues variables were not scaled, because this was not needed for model convergence.

| Parameter | Mean | SE | *P* value | Mean | SE | *P* value |
| --- | --- | --- | --- | --- | --- | --- |
| Wet pre-birth years | Males | | | Females | | |
| MDL | 2.963 | 2.117 | 0.162^a^ | 2.102 | 1.967 | 0.285^a^ |
| SAE_indvN-_*_A_*_<1_ | 2.277 | 3.568 | 0.523 |  |  |  |
| Intercept | -7.362 | 3.576 | 0.040 | -2.215 | 0.624 | <0.001 |
| Dry pre-birth years | Males | | | Females | | |
| MDL | -1.664 | 4.881 | 0.733^b^ | -1.075 | 1.549 | 0.487^b^ |
| SAE_indvN-_*_A_*_<1_ | -23.706 | 14.203 | 0.095 |  |  |  |
| Intercept | 4.974 | 7.787 | 0.523 | -2.111 | 0.436 | <0.001 |
