# Supplementary material for: A natural gene drive system influences bovine tuberculosis susceptibility in African buffalo: Possible implications for disease management
Source: PLoS One. 2019 Sep 4;14(9):e0221168. doi: 10.1371/journal.pone.0221168 (PMC6726202; doi:10.1371/journal.pone.0221168)
Supplement: S7 Table — (DOCX) [file pone.0221168.s015.docx]

S7 Table. Logistic regression between sex (dependent) and age and 3yr-pre-birth rainfall.

*N*_males(1)_ = 415, *N*_females(0)_ = 502, EPV = 207.5, Pearson correlation between main factors: *r* = 0.27 (all individuals). *N*_male-calves_ = 156, *N*_female-calves_ = 179, *N*_males>=2years_ = 259, *N*_females>=2years_ = 323.

Logistic regression analysis was implemented the `lme4' package (version 1.1.13) in R. Herd affiliation and sampling year were incorporated as a random intercept in a mixed modelling approach. To aid in regression model convergence, all continuous variables were scaled by subtracting the mean of each variable from each observation and dividing the result by the standard deviation of that variable.

| Parameter | Mean | SE | *P* value |
| --- | --- | --- | --- |
| 3yr-pre-birth rainfall (scaled), all  0-1-year old calves, ≥2-year olds | 0.227  0.756, 0.187 | 0.075  0.334, 0.080 | 0.0026  0.023, 0.019 |
| Age (scaled), all  0-1-year old calves, ≥2-year olds | -0.084  0.967, -0.147 | 0.072  0.998, 0.093 | 0.244  0.332, 0.114 |
| Intercept, all  0-1-year old calves, ≥2-year olds | -0.215  0.427, -0.177 | 0.080  0.763, 0.124 | 0.0073  0.576, 0.153 |
